# Exploring the potential and safety of quantum dots in allergy diagnostics

**DOI:** 10.1038/s41378-023-00608-x

**Published:** 2023-11-17

**Authors:** Milad Mohkam, Mohammad Sadraeian, Antonio Lauto, Ahmad Gholami, Seyed Hesamodin Nabavizadeh, Hossein Esmaeilzadeh, Soheila Alyasin

**Affiliations:** 1 Allergy Research Center, Shiraz University of Medical Sciences, Shiraz, Iran; 2 Institute for Biomedical Materials and Devices (IBMD), Faculty of Science, University of Technology Sydney, Sydney, NSW 2007 Australia; 3 School of Science, University of Western Sydney, Campbelltown, NSW 2560 Australia; 4 School of Medicine, University of Western Sydney, Campbelltown, NSW 2560 Australia; 5 Biotechnology Research Center, Shiraz University of Medical Sciences, Shiraz, Iran; 6 Department of Pharmaceutical Biotechnology, School of Pharmacy, Shiraz University of Medical Sciences, Shiraz, Iran; 7 Department of Allergy and Clinical Immunology, Namazi Hospital, Shiraz University of Medical Sciences, Shiraz, Iran

**Keywords:** Biosensors, Nanosensors, Chemistry

## Abstract

Biomedical investigations in nanotherapeutics and nanomedicine have recently intensified in pursuit of new therapies with improved efficacy. Quantum dots (QDs) are promising nanomaterials that possess a wide array of advantageous properties, including electronic properties, optical properties, and engineered biocompatibility under physiological conditions. Due to these characteristics, QDs are mainly used for biomedical labeling and theranostic (therapeutic-diagnostic) agents. QDs can be functionalized with ligands to facilitate their interaction with the immune system, specific IgE, and effector cell receptors. However, undesirable side effects such as hypersensitivity and toxicity may occur, requiring further assessment. This review systematically summarizes the potential uses of QDs in the allergy field. An overview of the definition and development of QDs is provided, along with the applications of QDs in allergy studies, including the detection of allergen-specific IgE (sIgE), food allergens, and sIgE in cellular tests. The potential treatment of allergies with QDs is also described, highlighting the toxicity and biocompatibility of these nanodevices. Finally, we discuss the current findings on the immunotoxicity of QDs. Several favorable points regarding the use of QDs for allergy diagnosis and treatment are noted.

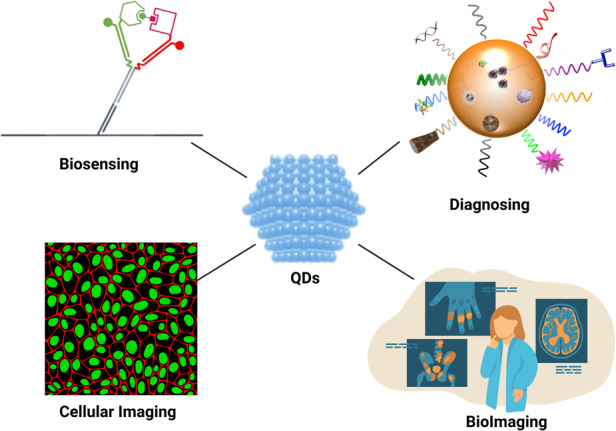

## Introduction

**Fig. 1:**
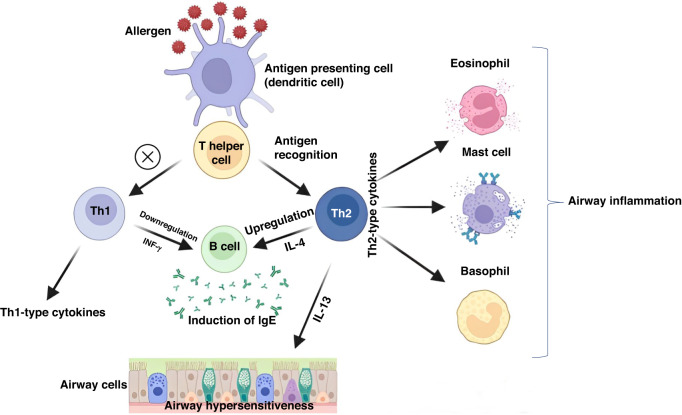
Pathogenesis of allergies and asthma: immune response mechanisms. Allergic reactions can be triggered by imbalances between pro-inflammatory Th2 cells and anti-inflammatory Th1 cells, resulting in an overactive immune response. B cells produce IgE antibodies that recognize and bind to allergens (e.g., drugs, foods), activating basophils, eosinophils, and mast cells. These cells release inflammatory mediators, such as histamine and leukotrienes, that cause symptoms such as itching, swelling, and difficulty breathing. The combination of these immune responses can lead to the development of asthma, a chronic respiratory condition characterized by airway inflammation and narrowing

Allergic disorders have become increasingly prevalent in recent decades, causing a significant financial burden on healthcare systems and decreasing overall well-being. One key factor contributing to the rise in allergic disorders is the Th1/Th2 imbalance, which plays a crucial role in the immune system’s response to allergens. This imbalance can lead to the production of excessive amounts of IgE, which can trigger allergic reactions (Fig. 1). Drug hypersensitivity reactions (DHRs), food allergies (FAs), and respiratory disorders are examples of allergic disorders often associated with this imbalance. DHRs are a typical example of a drug allergy, a severe condition that can result in life-threatening symptoms.

Similarly, respiratory disorders such as asthma and chronic obstructive pulmonary disease (COPD) are also associated with allergic responses and can difficulty in breathing and other symptoms^1^. Unfortunately, the variety of clinical manifestations and specific mechanisms of comorbidities result in poor diagnosis with low specificity or sensitivity, along with either ineffective or ineffective treatments to control the disease^2^. Therefore, improving the understanding of the underlying mechanisms of allergic disorders and developing more effective diagnostic and treatment options is essential in addressing the increasing prevalence of these conditions and mitigating their adverse impacts on individuals and society.

With the advent of nanomedicine in recent years, new possibilities have opened up in biomedicine, particularly in diseases such as allergies and in immunology. Nanotechnology allows the manipulation of materials with near-atomic dimensions and has applications in several scientific fields^3–5^. In this regard, several materials can be used for biomedical applications when functionalized with the desired ligands^6,7^.

**Fig. 2:**
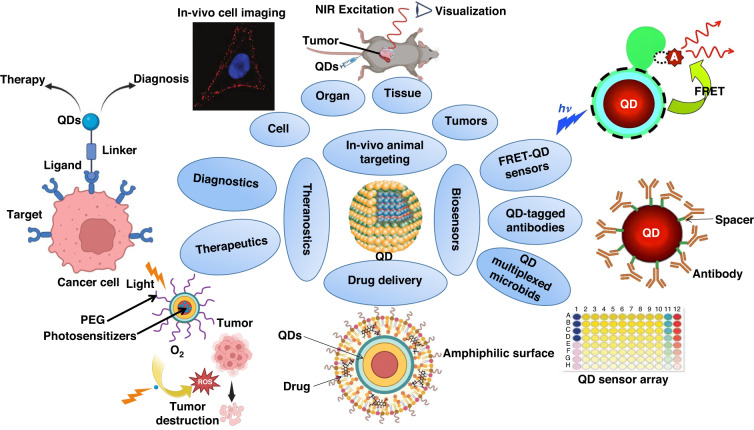
Top trending applications of QDs in biomedicine. The figure illustrates the multifaceted utility of quantum dots (QDs) in the field of biomedicine, showcasing several demonstrated applications in theranostic sciences. QDs have emerged as versatile tools with significant implications for drug delivery, biosensing, cancer immunotherapy, and gene therapy. These nanoparticles, with their unique properties, enable precise tracking and delivery of therapeutic agents, facilitate sensitive biosensing, and hold promise for innovative approaches in cancer immunotherapy and gene-based interventions

Quantum dots (QDs) are one of the most extensively studied types of nanoparticle substances since they possess specific photochemical, optical, and electronic properties. They were first reported in 1981 by Ekimov and Onushchenko, who discovered the quantum confinement effect, observing that CuCl crystals scattering within silicate glasses exhibit a size-dependent absorption pattern^8^. QDs were then used as a bioimaging material in biological systems^9^. Several applications of QDs in theranostic sciences have been demonstrated, including drug delivery, biosensing, cancer immunotherapy, and gene therapy (Fig. 2). Although QDs have potential and are expected to find application in the biomedical field, few have been approved for medical use; however, a small number of substances are in the clinical trial stage^10,11^.

Numerous interesting and promising QD applications have been demonstrated in vitro^11,12^. Nonetheless, problems associated with their in vivo toxicity still need to be addressed^13^. These applications involve attaching functional groups or ligands that target specific internal or external targets within tissues or cells. QDs can act as selective drug carriers that can discriminate among particular targets to reduce possible side effects. QDs can also enhance cargo protection, preventing destruction and improving water solubility for better drug delivery.

Furthermore, within the realm of photodynamic treatment (PDT), quantum dots (QDs), specifically carbon quantum dots engineered to imitate the structural characteristics of large amino acids, have emerged as a promising avenue for targeted tumor theranostics. This novel technique has substantial implications for its ability to address significant obstacles in photodynamic therapy (PDT), potentially improving its efficacy and mitigating undesirable effects. Carbon quantum dots have the ability to promote biocompatibility by structurally imitating large amino acids. This characteristic enables their seamless integration into medical applications and potentially mitigates the danger of toxicity. In addition, the ability of these structures to mimic the structural characteristics of cells could facilitate accurate targeting of specific cells, replicating the natural biological interactions of amino acids. This mimicry could enhance the accuracy of treatments while reducing harm to healthy tissues^14^. Because of their photoluminescent characteristics, these quantum dots can emit light at extended wavelengths, which may increase the production of reactive oxygen species (ROS) in the innermost layers of tumors.

These properties have the potential to improve treatment results. Carbon quantum dots possess a dual character, serving as therapeutic agents and prospective imaging tools, facilitating the real-time monitoring of therapy progress. Moreover, these quantum dots may demonstrate advantageous biodistribution and clearance characteristics due to their carbon-based composition, enhancing their overall safety^14^. For these reasons, QDs are suited for allergy diagnosis and promise to provide high specificity and sensitivity in vitro diagnostics, especially in IgE binding^15^. This review is focused on the latest QD applications to in vitro allergy diagnostic technologies, their toxicity issues, and innovative treatments.

## Quantum dot properties

**Fig. 3:**
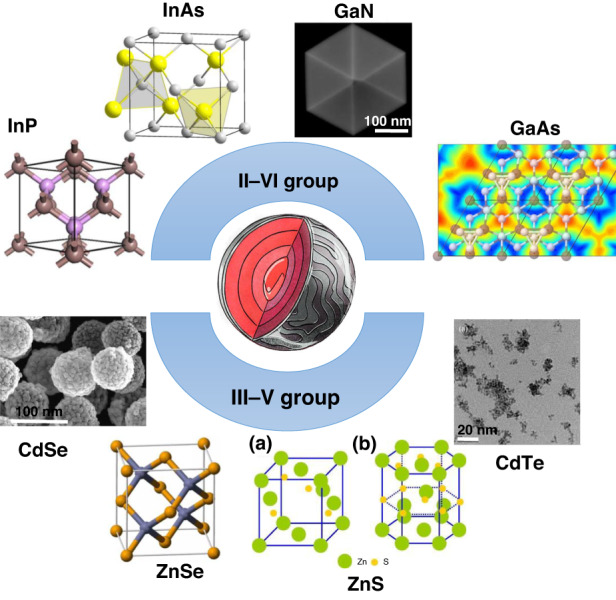
The 3-D chemical structure of representative examples of group II–VI and III–V semiconductors with higher atomic masses that can act as QDs. The SEM micrographs show the morphologies of GaN, CdSe, and CdTe. ZnS quantum dots are mainly available in the blend phase (**a**) and the wurtzite (**b**) phase

QD particles comprise a semiconductor core enclosed in a semiconductor compound shell. The core is a metalloid III–V or II–VI semiconductor. A semiconductor moiety is an electrical conductor with properties between those of electrically conductive and non-conductive materials. Some of these III–V compounds include indium arsenide (InAs), indium phosphide (InP), gallium nitride (GaN), and gallium arsenate (GaAs)^16^. Representative group II–VI compounds include zinc selenium (ZnSe), zinc sulfide (ZnS), cadmium tellurium (CdTe), and cadmium selenium (CdSe)^17^. Additionally, some investigations suggest that combining elements with higher atomic masses, such as CdSe/ZnTe or CdTe/CdSe, can produce QD behavior (Fig. 3).

The properties of quantum dots are determined by their size, shape, composition, and surface chemistry. Some fundamental properties of quantum dots include emission wavelength, quantum yield, stability, toxicity, and biodegradability (Table 1).

**Table 1:** Material properties of different quantum dot systems

Material system	Emission wavelength	Quantum yield	Stability	Toxicity	Biodegradability
Metal chalcogenides	Visible to near-infrared	High (>80%)	Moderate to high	High	Low
Metal halides	Visible to near-infrared	High (>80%)	Low to moderate	Moderate to high	Low to moderate
C-dots	Ultraviolet to near-infrared	Low to high (<10% to >80%)	High	Low	High
Si-dots	Visible to near-infrared	Low to high (<10% to >80%)	Moderate to high	Low	High

**Fig. 4:**
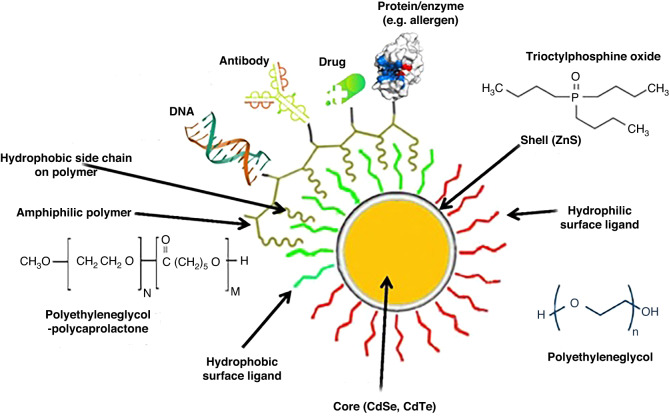
Functionalization of QD surfaces with diverse molecules. The surface can be decorated with a wide range of molecules, such as hydrophilic, hydrophobic, and amphiphilic molecules (typical coating molecules are displayed), which can then be further connected to drugs, proteins, antibodies, and other compounds

Incorporating functional groups into the core/shell of QDs can create novel bioactive compounds for usage in biology^18,19^. Numerous biological molecules can attach to the surface of the QD shell, including peptides, proteins, and lipids. It is possible to cap the thiol groups by forming a covalent bond with the surface shell to make QDs more water-soluble^12,20^. QD surfaces can also be coated with polymers such as polylactide co-glycolides, polymethyl methacrylate, polyvinyl alcohol, and polyoxometalate^21^. These polymers are sometimes applied alone as biosensors for detecting and measuring medicines and in-blood factors^22,23^. Surface-modified QDs can treat, diagnose, or prevent specific diseases within the body (Fig. 4)^24^. Various approaches are available for functionalizing the outer shell of QDs, including physical adsorption (physisorption), electrostatic interactions, covalent bonds, and multivalent chelation. Additionally, surface attachment can substantially affect the size of QDs^25,26^.

It has been shown that the particle size of QDs is related to the emission wavelength, so by modifying the particle size, a wide range of spectra (UV-IR) can be produced. This property has been exploited using QD signal multiplexing for tracking and imaging multiple molecular targets with high resolution. In contrast, organic dyes generally have broad emission bands, which considerably increase the difficulty of recognizing various signals. In addition, organic dyes have strong background signals, which greatly decrease detection sensitivity and thus probe utility^19,27,28^. Accordingly, QDs can reduce background signals while still being tuned for a desired spectral emission.

## Biomedical application of quantum dots

Quantum dots (QDs) have been employed for biological applications since 1998 when they were initially conjugated with a biomolecule. Subsequently, fluorescent bioimaging has emerged as a potent methodology, enabling researchers to visualize the internal structures of cells and molecules^29^. This section provides a comprehensive overview of recent and groundbreaking investigations employing QDs for biological imaging, drug delivery, and photodynamic therapy applications.

### Imaging

Quantum dots exhibit numerous advantageous characteristics for optical applications, including elevated luminosity, enhanced stability, and heightened efficacy^30,31^. Furthermore, the coloration of these entities can be altered by modifying their dimensions or the composition of their core material^32^. For example, size alteration within the range of 2–10 nm in CdSe QDs can generate various hues within the visible spectrum (400–600 nm)^33^. Likewise, their fundamental composition can influence the chromatic and dimensional characteristics of quantum dots (QDs). As an illustration, QDs possessing a CdS core exhibit dimensions ranging from 1 to 6 nm and emit light within the ultraviolet‒visible (UV‒VIS) spectrum. Conversely, QDs featuring an InAs core of equivalent size emit light within the infrared (IR) range^34^. The characteristics above render quantum dots highly advantageous for fluorescent bioimaging, a methodology employed to visualize biological structures through light utilization^35^. QDs can be utilized for cellular imaging, enabling the visualization of cells and their constituents. The facile cellular uptake of QDs can be attributed to their small size. Subsequently, the specimens can be optically excited by light and exhibit fluorescence, which can be discerned through microscopes or confocal laser scanning microscopy (CLSM). QDs possess a distinctive characteristic known as blinking, wherein they randomly switch between active and inactive states. This technique facilitates the identification of individual QDs and enables the tracking of individual molecules within cellular environments^36^. QDs can potentially be employed to visualize internal organs and tissues within live animals. The attachment of specific molecules to QDs can facilitate their binding to particular organs or tissues. Subsequently, the visual detection of QDs can be achieved through cameras or scanners^37^.

#### Drug delivery

The use of nanoparticles in medication delivery studies has notably grown in recent years^38^. Several experiments have established the capacity of QDs to be delivered intravenously and then distributed to essential organs such as the liver and kidneys^39^. This result means that QDs have the potential to serve as carriers for drugs, facilitating their delivery to specific target regions while maintaining their stability until arrival. QDs exhibit intense fluorescence, thereby aiding the tracking of drug distribution within the body by researchers.

Furthermore, QDs can enter diverse cellular types and localize within distinct cellular compartments, including lysosomes^40,41^. This characteristic has proved helpful for quantum dots (QDs) that function as carriers for pharmaceuticals that must be released within acidic settings, such as the lysosome. Hence, QDs display tremendous promise in the realm of pharmacological medicine delivery. Nevertheless, several difficulties must be conquered, including issues related to toxicity and selectivity.

Cadmium-based quantum dots are one of the most prevalent kinds of quantum dots. They are renowned for their excellent fluorescence characteristics with both high brightness and remarkable stability^42^. This property renders them well-suited for constructing a nanocarrier system that can be optically traced. In a recent effort, a unique system was designed combining CdSe quantum dots and the chemotherapeutic drug doxorubicin (Dox) enclosed within phospholipid micelles. Phospholipid micelles operate as microscopic vesicles capable of encapsulating CdSe QDs and Dox, thereby improving their water solubility^43^. The CdSe quantum dots serve as a fluorescent probe for visualizing the distribution of the drug throughout the organism.

In contrast, Dox is a chemotherapeutic agent with lethal capabilities selectively targeting malignant cells. The present study successfully supported the targeted delivery of Dox to HeLa cells, a specific type of cervical cancer cell. This observation shows that applying the CdSe QD-Dox micelle system holds promise in medicine administration. Nevertheless, there remain outstanding questions that must be addressed before the adoption of this technology in actual situations. The precision of this method in preferentially targeting HeLa cells while limiting harm to normal cells remains unknown. Hence, it is critical to undertake more studies to identify this method’s comparative effects on distinct cell types, encompassing both malignant and noncancerous cells. In addition, it is vital to conduct experimental trials on live animals to evaluate the functionality and efficacy of this system under authentic conditions. One of the critical problems related to materials containing cadmium is their high toxicity, which results from the release of cadmium ions into the human body^44^. Hence, more inquiry is necessary to examine the safety and efficacy of the CdSe QD-Dox micelle system.

Compared to quantum dots (QDs) consisting exclusively of a core, encapsulated cadmium-based QDs have more promise for usage in biology. The outer shell, which serves as a protective barrier, is critical in restricting the escape of cadmium ions^45^. A research project was designed to deploy CdSe/CdS/ZnS quantum dots (QDs) as carriers for Dox, specifically targeting rat alveolar macrophages. The aim was to maximize the efficacy of drug delivery to targeted lung cells while reducing the possibility of a protracted inflammatory reaction. The results of the study suggested that the combination of CdSe/CdS/ZnS quantum dots (QDs) with Dox increased the transport of Dox to alveolar macrophages in rats. Furthermore, it was revealed that the quantum dots (QDs) were distributed in the cytoplasm, whereas doxorubicin (Dox) was limited to the nucleus^46^. This result shows that the release of Dox from the QDs upon cellular absorption was successful.

The integration of graphene quantum dots (GQDs) offers a promising approach for developing a medication delivery system utilizing quantum dots (QDs). The coupling of graphene quantum dots (GQDs) with doxorubicin (Dox) and the selective targeting molecule arginine-glycine-aspartic acid (RGD) was conducted within the framework of this delivery architecture. The study’s findings suggest that the impact of unbound graphene quantum dots (GQDs) on the viability of prostate cancer cell lines (DU-145 and PC-3) is negligible at concentrations below 100 µg/mL. According to a study conducted by Qiu et al., there was a marginal decline in cell viability when exposed to a higher dose of 400 µg/mL^47^. Furthermore, the release of Dox from graphene quantum dots (GQDs) exhibited a pH-dependent response. The release of Dox was observed to occur gradually at neutral pH, but rapid release occurred at pH 5^47^. pH-sensitivity has shown potential for controlling the release of medicinal drugs within cells. The findings of this investigation indicate that graphene quantum dots (GQDs) demonstrate minimal toxicity, presenting promise for a meticulously regulated drug delivery platform.

There has recently been a considerable surge of interest among researchers in a newly discovered category of quantum dots referred to as carbon dots. This heightened attention can be attributed to the remarkable lack of toxicity associated with these carbon dots. The utilization of fluorescent carbon dots in conjunction with hyaluronic acid and carboxymethyl chitosan (CDC-H) ligands was employed by a team of researchers^48^. The ligands exhibit a high binding affinity toward CD44 receptors, which are overexpressed in several cancer cell types. A recent innovation has successfully devised a drug delivery system that demonstrates the ability to effectively provide doxorubicin (Dox) to specific cancer cells while ensuring the preservation of traceability. The findings derived from a 3-(4,5-dimethylthiazol-2-yl)-2,5-diphenyltetrazolium bromide (MTT) assay demonstrate the absence of cytotoxicity of the (DOX-CDC-H) complex to NIH3T3 fibroblasts.

In contrast, the molecule resulting from the combination of DOX-CDC-H exhibited cytotoxicity against two distinct breast cancer cell lines, specifically MCF-7 and 4T1^48^. Notably, the observed complex showed a stronger effect on 4T1 cells than MCF-7 cells, possibly attributable to the upregulated expression of CD44 in 4T1 cells. On the other hand, free Dox exhibited indiscriminate cytotoxicity across all the cell lines that were investigated^48^. Hence, it may be deduced that within an artificial laboratory setting, the (DOX-CDC-H) complex demonstrates selective eradication of breast cancer cells expressing CD44 while remaining harmless to healthy fibroblasts. The complex’s notable level of selectivity renders it a highly compelling contender for utilization in medicine delivery.

In a similar investigation, researchers employed a distinctive approach to generate carbon-based fluorescent graphene nano-biochar (NBC) that could be readily marked with various targeting ligands. This nano-biochar facilitated the targeted administration of the anticancer compound DHF (5,5-dimethyl-6a-phenyl-3-(trimethylsilyl)-6,6a-dihydrofuro[3,2-b] furan-2(5H)-one) to neoplastic cells. The drug delivery approach utilizing NBC enhanced the solubility of DHF. In addition, the incorporation of targeting ligands, namely, riboflavin (R) and biotin (B), significantly increased the internalization of NBC by A549 lung cancer epithelial cells^48^. The evidence discussed above demonstrates the attributes of NBC-TL as a robust drug delivery method for medicines with limited solubility.

### Photodynamic therapy

Photodynamic therapy (PDT) has been recognized as a promising modality in the fight against cancer, signifying an increasingly prominent approach^49,50^. The treatment technique under consideration combines photophysical and photochemical processes to achieve a result of physiological significance^51^. QDs in photodynamic therapy have been extensively employed due to their capacity to produce singlet oxygen when exposed to light through inherent photosensitizers. Water-soluble nanocomposites have been effectively synthesized through the amalgamation of CdSe/ZnS QDs with hydrophobic tetraphenyl porphyrin (TPP) molecules encapsulated within chitosan. The innovative technology exhibited a notable mean efficacy of 45% in generating singlet oxygen, which can be attributed to intracomplex Förster resonance energy transfer with TPP^51^.

The procedure entails the photochemical interaction between an excited photosensitizer and cellular substrates or molecular oxygen, eradicating malignant cells. In its original state, the photosensitizer possesses a low-energy molecular orbital that accommodates a pair of electrons with opposite spins. When subjected to illumination, an electron experiences a transition to atomic orbitals with higher energy levels while retaining its spin. This process results in the creation of the singlet excited state^52^. However, the photodrug’s ability to interact with cellular substrates is hindered by the short duration of the singlet excited state, typically lasting only nanoseconds to picoseconds.

In an excited state, the photosensitizer exhibits the capacity to engage in two distinct processes: fluorescence, characterized by the emission of light energy and subsequent restoration to its initial state, or nonradiative decay, involving the release of heat energy through internal conversion (IC). An extensive examination of progress in photosensitizers is needed, spanning from their initial forms to the contemporary third-generation iterations, alongside the enhancement of delivery mechanisms, manipulation or inhibition of immune responses, synergistic treatment approaches and other pivotal facets of photodynamic therapy. The use of recently created quantum dots is a burgeoning area within photodynamic treatment (PDT). The QDs examined in this research have significant advantages that effectively address the limitations associated with conventional photodynamic treatment (PDT) agents. The abovementioned benefits include outstanding photostability, heightened quantum yield, and notable transition dipole moment^53,54^.

In addition, the core’s inherent photophysical characteristics and solvent capacities can be precisely tailored to fulfill specific demands by manipulating its dimensions and composition. This can create a substantial surface area for binding biomolecules, such as peptides and antibodies^55^. Nevertheless, the application of QDs alone in photodynamic therapy (PDT) yielded less than optimal outcomes. However, QDs have the potential to actively engage in fluorescence resonance energy transfer (FRET) as energy donors, a methodology extensively utilized by several researchers in photodynamic treatment (PDT)^55^. Förster resonance energy transfer (FRET) can be recognized by the decrease in fluorescence released by the donor particles and the corresponding increase in fluorescence emitted by the acceptor particles. While quantum dots (QDs) do not exhibit an intrinsic capability for generating singlet oxygen in isolation, their integration with natural dyes significantly enhances singlet oxygen production, as evidenced by the quantum efficiency^55^. Quantifying the distance between QDs and photosensitizers (PSs) in nanometers is paramount in determining Förster resonance energy transfer (FRET). Their association can be preserved through the production of noncovalent complexes or the establishment of covalent bonds^55^. Several surface modification techniques have been utilized to address the challenge of maintaining the stability of QDs in biological environments. Amphiphilic compounds are commonly employed to encapsulate QDs in a broad context. The hydrophobic component of the molecule surrounds the hydrophobic QD, thereby facilitating its dispersion in a solvent. This encapsulation methodology is widely adopted due to its effective resolution of concerns related to luminescence quantum yields and colloidal stability^55^.

The size of particles plays a crucial role in determining the appropriateness of quantum dots (QDs) for applications in diagnostics and therapeutics. To ensure successful penetration of biological barriers such as the alveolar-capillary barrier, blood‒brain barrier, gastric and cutaneous barriers, and renal filtration barrier, a QD must possess a core with a minimum diameter of 8 to 10 nm. It is essential to acknowledge that the necessary diameter tends to grow when considering surface features^56^. Comprehensive research on the potential toxicity of quantum dots (QDs) is of utmost importance for their integration into clinical settings for human use. The process mentioned above inherently requires a significant amount of time^57^.

According to a recent study, the efficacy of photodynamic therapy in targeting cancer cells has been notably enhanced through the utilization of carbon dots derived from curcumin and folic acid. This work presents conjugated carbon dots (CCDs), a newly discovered photosensitizer that can demonstrate two-photon activity^58^. The experimental findings show the generation of highly reactive oxygen species (ROS) that exhibit potent cytotoxic effects, particularly by selectively targeting the nucleus for photodynamic treatment (PDT)^58^. The CDcf system revealed notable interaction with malignant cells, leading to conspicuous nuclear localization by a mechanism facilitated by folate receptors. Subsequently, two-photon excitation resulted in increased reactive oxygen species (ROS) generation within the nucleus, amplifying the efficacy of photodynamic treatment (PDT)^58^.

Consequently, the effectiveness of eliminating cancer cells has been dramatically enhanced by directly targeting their DNA. The inherent capacity of carbon dots (CDs) to produce reactive oxygen species (ROS) and selectively localize in the nucleus has enabled an integrated framework to advance a versatile dual-photon active nanoformulation^58^. The invention of CDcf introduced a new methodology that effectively and efficiently directs and administers PDT chemicals. Moreover, it is essential to highlight that this specific technology demonstrates significant potential for future advancements, as it can enhance nanoprobe effectiveness by incorporating customized therapeutic and diagnostic functionalities^58^.

## Value of in vitro diagnostic techniques in allergy

It is necessary to conduct a comprehensive examination to diagnose allergic diseases and distinguish those caused by T cells or specific immunoglobulin E (sIgE), particularly in DHRs^59,60^. Various factors may trigger IgE-mediated allergic reactions, including food allergens, aeroallergens, and drugs^61,62^. The drugs require a carrier protein to reach the required size to engage the immune system and activate the associated responses. In this context, carriers have been shown to affect IgE detection, suggesting the need for an appropriate adduct to be included in the diagnostic methods^63,64^.

It has been shown that superlative techniques in immunoassays are based on identifying mediator liberation during the acute phase of a reaction, including histamine, leukotrienes, or tryptase liberation. The in vitro determination of the wrongdoer drug/allergen is also intended to identify the wrongdoer drug/allergen using functional basophil activation tests (BAT) and immunoassays to determine the concentration of sIgE^65,66^.

In immunoassays, serum sIgE quantification against allergens is the most frequently used method, which can be multiplex or singleplex^61^. Several singleplex assays with the advantages of adaptability and automation are thus available today and can be applied to various samples, including extracts and recombinant and purified native allergens^67^. On the other hand, multiplex arrays can provide extensive allergen profiles (more than 100) and a wide variety of samples, such as extracts, recombinant allergens, and purified native allergens. The advantage of these systems is that they provide information for the sensitization of a considerable assortment of chemicals with minimal serum amounts. Despite the high sensitivity of these systems, the specificity is not optimal: they cannot differentiate clinically relevant IgE due to the similar cross-reactivity of allergens and cross-reactivity of carbohydrate determinants (CCDs)^68,69^.

A combination of an electrochemical sensor and immunoassay can be used to detect the specific binding reaction between the antibody and antigen, converting these data directly into electrical signals and enabling the rapid recognition of particular disease markers in liquid samples. To this end, a high-sensitivity electrochemical sensor was developed that recognizes eosinophil cationic protein (ECP), an allergic rhinitis biomarker, by using a conductive carbon electrode that was decorated with semiconductor colloidal quantum dots (CQDs)^23,70^. Lead sulfide (PbS) CQDs exhibit excellent adhesion to carbon electrodes due to their remarkable capacity to enrich biomolecules and enhance signal transduction. Differential pulse voltammetry (DPV) was also combined with immobilization of the ECP antibody to reduce background current during electrochemical performance tests. These modifications led to the accurate measurement of ECP antigens at various concentrations. Electrochemical sensors can detect ECP antigens within 30 s. Their thresholds are 0.508 ng/mL, comparable to those of commercial enzyme-linked immunosorbent assay (ELISA) tests, which can detect a minimum of 0.39 ng/mL but require an operation time of 1–5 h at a high cost^71^. These results indicate that electrochemical sensors are noninvasive and convenient, have superior sensitivity, and are suitable for monitoring diseases such as allergic rhinitis.

A solid-phase immunoassay for DHRs was developed using drug-carrier conjugates to detect serum-drug-sIgE^72^. However, there are several problems with currently available immunoassays. In-house radioimmunoassay (RIA) and commercial ImmunoCAP-FEIA (fluoro-enzyme immunoassay) have inherently inadequate detection limits, primarily owing to insufficient levels of drug-sIgE or the use of incorrect drug adducts or drug metabolites^73^. Despite recent progress, no commercial assay systems based on QDs are available.

## Detection of allergen-specific IgE

QD nanostructures have remarkable physicochemical characteristics. For example, they can be tuned and controlled accurately, paving the way for in vitro diagnosis by enhancing specific IgE binding, imitating carrier proteins, or improving the recognition of signals^73^.

Many quantum dots have been used for improving allergy diagnostic tests, including metallic QDs and graphene-based QDs^73^. Most of these studies focus on determining sIgE levels in immunoassays to allergens. Concerning immunoassays for the determination of sIgE, quantum dots have recently been tested as detection instruments and solid support for capturing allergens and antibodies with the advantage of improving and intensifying the measurement signal via immunochromatographic techniques, electrochemical techniques^74^, electrochemical techniques^75,76^ or dual polarization interferometry^77,78^. However, due to the novelty of QDs and legal regulations, no commercial device has yet been developed for the abovementioned techniques. There are also reports of QD-based nano-biosensors being able to detect immunologic consequences of viral diseases such as COVID-19^26,79,80^.

QDs are intrinsically electroactive, enhancing the analytical performance of electrochemical IgE detection. The electrical signal generated by an antibody and an antigen is measured in this method. Due to their distinctive characteristics as electro-chemosensors, the deposition of QDs onto the electrochemical electrode significantly enhances signal amplification^81^. For instance, the deposition of CdSe/ZnS QD-functionalized MoS_2_ could be applied for efficient signal amplification to determine IgE concentrations, as reported by Liu et al.^82^.

The immunochromatographic assay is considered one of the most effective techniques for detecting IgE. The detection of low concentrations of IgE in sera was enabled by functionalizing immunochromatographic vessels with QD metal substrates^83^. Therefore, this method is faster and less labor-intensive than using a conventional ELISA kit (~2 h). In contrast, a commercial immunochromatographic test, the Allergy Lateral Flow Immunoassay (ALFA Total IgE test), invented by Neuss Corporation, Germany, allows quantitative detection of IgE by revealing colored lines^84^. More precisely, the MQTE 1 immunochromatographic test was developed by Milenia Biotec (Giessen, Germany) and offered quantitative results for total IgE in serum, but with a relatively low threshold measurement of 30 kU/L compared to ELISA kits (5 kU/L)^74^. The incorporation of fluorescent markers may reduce detection limits, along with a reduction in matrix impact. This fact suggests that QDs are appropriate fluorescent markers for immunoassays^85–87^. In addition to their stable, symmetrical, and narrow emission properties, QDs are resistant to photobleaching and offer better optical performance than organic fluorescent labels^88,89^. For instance, Berlina et al. developed an immunochromatographic assay that recruited CdSe/ZnS QDs as fluorescent labels for detecting and quantifying total IgE in human serum with a low threshold of 5 kU/L, similar to the commercial ELISA kit^74^. More interestingly, Zhao et al. developed a novel lateral flow immunoassay that employed CdSe/ZnS quantum dot nanobeads as fluorescent labels to detect specific IgE antibodies against *D. pteronyssinus* allergen 1 (nDer p 1) with a low threshold measurement of 0.2 kU/L, which enables the detection of lower levels of IgE than a commercial ELISA kit^90^.

QDs can further amplify signal detection to achieve much higher sensitivities by combining them with detecting molecules, such as aptamers, enzymes, or antibodies^81^. For instance, Shi et al. developed an electro-chemiluminescent aptasensor based on CdS-MoS_2_ QDs and DNAzyme for high levels of specificity for human immunoglobulin E^81^. Similarly, Liu et al. harnessed a specific IgE binding aptamer created from CdSe/ZnS-functionalized MoS_2_ using HP-catalyzed biocatalysis (BCP) for signal quenching^82^. Therefore, QDs can bind to sIgE and enhance the detection signal by coupling detection molecules, such as aptamers, antibodies, or enzymes, to improve sensitivity. In this sense, further evaluations may be required in some complex systems, such as human blood samples, which may face issues due to matrix effects that may adversely affect the trial results. Recently, an antifouling sensing interface was developed for an electrochemical biosensor based on the self-assembly of IgE aptamers and zwitterionic peptides on macroporous gold substrates to resolve these limitations. In this manner, the zwitterionic peptide diminishes fouling effects and nonspecific adsorption. In contrast, the aptamer’s high specificity, as well as the superior surface area resulting from the porous morphology, allows it to demonstrate superior selectivity and sensitivity with regard to IgE, thereby allowing it to measure IgE in biological specimens 94 sensitively.

## Detection of sIgE in cellular tests

Specific activation of effector cell assessments is at the core of an alternative assay for evaluating IgE-mediated allergic reactions. For example, the basophil activation test (BAT) has been found to be beneficial for rapidly detecting hypersensitivity reactions, including those to Hymenoptera venom, drugs, and food allergies^65^. Nevertheless, there is a need to reduce labor requirements, enhance performance, and improve the cost-effectiveness and user-friendliness of these assays. Using QDs as a fluorescent probe to label allergens is a new technique for evaluating BAT, where the activation biomarkers can be gated on cell surfaces with various labeled allergens. This strategy allows multiplex BAT evaluations involving multiple allergens with a significantly reduced number of examination probes. A previous report showed that conjugated QDs containing multiple allergens (Api m 1 (phospholipase A2) + Api m 2 (hyaluronidase)) could effectively dose-dependently stimulate basophils in patients allergic to bee venom^91^. Therefore, QDs in BAT can detect IgE with greater precision due to a narrower and brighter emission spectrum and a higher range of excitation wavelengths compared with more conventional dyes/fluorophores.

## Detection of food allergens

One attractive application of quantum dots is the identification of trace allergens in multifaceted food matrices^92^. There is growing interest in applying QDs to improve signal recognition in biosensors for food analysis and related applications. For example, QDs can improve ELISA performance by providing narrow emission bands and high brightness as fluorophores^92,93^. In this regard, a sensitive fluorescent sandwich ELISA was successfully developed by quenching thiolated CdTe quantum dots in the presence of hydrogen peroxide as the fluorescent signal output through catalase-mediated fluorescence quenching. The sensitivity of this system (for bovine β-lactoglobulin detection) is approximately 16-fold less than that of horseradish peroxidase-based assays^94^. In parallel, a quantum dot nanobead-based immunosorbent assay (QB-ELISA) based on CdSe/ZnS has been developed for quantifying glycinin in soybeans and soybean products. A sevenfold increase in glycinin detection sensitivity was achieved with this method while reducing the detection time to one-third^95^. More recently, an immunoassay based on fluorescence resonance energy transfer (FRET) was developed for the detection of arginine kinase, a major allergen in shrimp, using InP/ZnS quantum dots with high sensitivity and selectivity^96^. Therefore, QD-based detection systems have a threshold that aligns with international legislative standards and are suitable for commercial applications.

## Allergic disease treatment

An allergen or drug avoidance strategy is the first step in managing allergic diseases; unfortunately, this strategy may not always work, especially in food allergies with ubiquitous and hidden sources of allergens, as well as in DHRs when patients cannot replace the responsible drug. Allergen immunotherapy is effective for various pathologies, such as respiratory diseases, insect venom allergies, and food allergies. AIT is the only treatment capable of interfering with the etiology of allergic diseases^97–99^, but certain issues require attention, including the possibility of causing systemic reactions, particularly with FA, and the standardization of allergen preparations, which must include the concentration of allergens and the number of allergenic proteins. Various tailored therapeutic approaches are employed to address this limitation^99,100^, which rely on using hypoallergenic variants or B and T-cell peptides capable of reducing the likelihood of an allergic reaction. However, they may also exhibit low immunogenicity, and to achieve a more effective immunological response and reduce adverse reactions associated with conventional immunological therapy, platforms that encompass all the components required for effective interaction with particular cells within the immune system would be essential.

As a general principle, quantum dot technology may be helpful for immunotherapy, as QDs can function as adjuvants^101^. When compared to conventional adjuvants, QDs provide the advantage of protecting allergens against hydrolysis or enzymatic breakdown, preventing the identification of allergens via IgE on effector cells, such as basophils or mast cells, and ensuring that the allergens are presented optimally, eliciting an immunogenic response without a significant allergenic effect^102^. In addition, QDs may also facilitate a ‘depot’ effect, enabling a controlled concentration of the allergen to be exposed to the immune system for an extended time. In most cases, larger particles can cause this as they become trapped in tissues near the injection site, thus increasing local antigen retention^103^. A potential adjuvant is formed when QDs interact with antigen-presenting cells (APCs), including DCs, to modulate the immune response. This system produces a more controlled effect by functionalizing them with ligands, making them suitable platforms for tuning their physicochemical properties and facilitating selective drug delivery to target cells^104,105^.

As part of a study on allergies to 1,4-fluoro-2,4-dinitrobenzene, tiny quantum dots with a ZnS/CdSe shell/core with a negatively charged surface (using glutathione as the surface) demonstrated immunosuppressive influences by reducing allergies to this compound. In this context, QDs can affect the skin by penetrating it^106^.

Inflammatory diseases, such as allergies, are associated with oxidative stress, which is crucial in their development^107^. Gao et al. developed defect-containing Ag–In–S/ZnS quantum dots (AIS/ZnS QDs) that can scavenge oxygen-induced radicals. The intrinsic defects in these quantum dots and many surface functional groups result in the remarkable elimination efficiency of oxygen-derived free radicals in vitro. The AIS/ZnS QDs can effectively remove extra ROS triggered by either lipopolysaccharide (LPS) or H_2_O_2_. As a result, macrophages are protected from oxidative damage caused by ROS. Furthermore, after LPS-induced inflammation, macrophages demonstrated protective anti-inflammatory activity by suppressing the expression of pro-inflammatory cytokines (e.g., TNF-α and IL-6). These outcomes suggest that AIS/ZnS QDs may effectively treat inflammation, including allergic responses caused by ROS^108^.

Traditional Chinese medicine and carbon dots (CDs) were combined to treat allergic reactions. Kong et al. created a method to prepare water-soluble CDs using aqueous Scutellariae Radix Carbonisata (SRC) extracts. In vitro studies in a C48/80-induced RBL-2H3 cell model revealed notable anti-allergy effectiveness of the SRC-CDs that could offer new technical approaches for the analysis of charcoal drugs, leading to a better understanding of their potential applications in biomedicine, including allergic disorders^109^.

As discussed in the previous sections describing the applications of quantum dots (QDs) in the field of allergic illness diagnostics and treatment, it is clear that QDs have unique properties that make them particularly well-suited for these purposes.

### High brightness and photostability

QDs exhibit notable characteristics such as high luminosity and resistance to photodegradation, which can be attributed to their substantial absorption cross-sections and minimal nonradiative decay rates. This implies that QDs can produce strong and consistent fluorescence signals when subjected to continuous stimulation. This characteristic can enhance the precision and sensitivity of allergen identification, immune cell analysis, and immune response imaging.

### Tunable emission spectra:

QDs have tunable emission spectra, which are contingent upon their dimensions, morphology, and chemical makeup. This means that QDs can emit a diverse range of light wavelengths, adjusted by altering their physical or chemical characteristics. This property facilitates the simultaneous detection and imaging of numerous targets or processes within biological systems, a technique known as multiplex detection.

### Surface functionalization

QDs can undergo surface functionalization, enabling them to be modified with a range of biomolecules or biomimetic materials. This modification process can potentially improve the biocompatibility, stability, solubility, and specificity of QDs. This implies that QDs can be coated or conjugated with various biomolecules, such as antibodies, peptides, aptamers, or small molecules that can specifically recognize and bind to particular receptors or antigens present on the surface of target cells or tissues.

### Stimuli-responsiveness

QDs include stimulus-responsive characteristics that allow them to react to both external and internal stimuli, including but not limited to light, heat, pH, enzymes, and ultrasound. This means that QDs can be activated or deactivated by the application of various stimuli. Consequently, this ability enables the manipulation of their optical or electronic characteristics and their controlled release of medications or genes.

## Delivery and release methods of quantum dots

Quantum dots (QDs) possess various advantageous characteristics that make them suitable for diagnosing and treating allergic illnesses. These advantages include their high brightness and photostability, tunable emission spectra, capacity for surface functionalization, biocompatibility, and low toxicity. To fully harness the potential of quantum dots (QDs) in biomedical applications, it is imperative to devise efficient and secure strategies for the delivery and subsequent release of QDs to specific cells or tissues^110^.

Many approaches to the administration and dissemination of quantum dots (QDs) exist, contingent upon their specific characteristics, such as type, size, shape, surface chemistry, and functionalization. Additionally, the selection of these strategies is influenced by the intended target cells or tissues, the desired mechanism of action, and the potential adverse effects^55^. Several often-used approaches are described below.

### Direct injection

Quantum dots (QDs) can be introduced into the circulatory system or specific tissues using direct injection methods, including a syringe or needle. This technique is simple and expedient; nonetheless, it has the potential risk of undesired aggregation or dispersion of quantum dots within the organism, alongside potential concerns regarding their toxicity or immunogenicity. As an illustration, the intravenous administration of CdSe/ZnS quantum dots (QDs) in mice led to the rapid elimination of QDs from the systemic circulation and subsequent accumulation in the liver and spleen^111^. Direct injection of CdTe-QDs into rats induced oxidative stress and inflammation in the liver and kidney^112^. Direct injection of CdS QDs into mice triggered immune responses and the formation of granulomas at the injection site^113^.

### Encapsulation

Quantum dots (QDs) have the potential to be encapsulated within biodegradable or biocompatible materials, such as polymers, lipids, proteins, or silica, resulting in the formation of nanoparticles or microparticles. These particles can protect quantum dots (QDs) by preventing degradation or aggregation and enhancing their stability and solubility. Additionally, these particles can improve the biocompatibility and specificity of QDs^114^. The particles can be introduced through injection or oral, intranasal, or topical administration routes. The controlled release of quantum dots (QDs) can be achieved by applying many stimuli, including variations in pH levels, temperature, light exposure, enzymatic activity, or ultrasound^114^. Several examples of encapsulation methods include the following:

#### Polymer encapsulation

Polymer encapsulation is the application of polymers, such as polyethylene glycol (PEG), poly(lactic-co-glycolic acid) (PLGA), or chitosan, to coat quantum dots (QDs) to create core-shell nanoparticles. Polymer encapsulation has been shown to enhance the water solubility, biocompatibility, and stability of quantum dots (QDs). Additionally, this technique offers further customization by introducing functional groups^115^. The process of polymer encapsulation offers the potential for regulating the release of quantum dots (QDs) by adjusting the degradation rate of the polymer matrix. This technique has been explored in the hydrogel encapsulation of QDs^116^.

#### Lipid encapsulation

Quantum dots (QDs) have the potential to be integrated into lipid-based architectures, including liposomes, micelles, and solid lipid nanoparticles. Lipid encapsulation has been shown to enhance the biocompatibility and bioavailability of quantum dots (QDs) while facilitating their transportation through biological barriers^117^. Lipid encapsulation can facilitate the responsive release of quantum dots (QDs) by capitalizing on the phase transition or fusion phenomena shown by lipid bilayers^118^.

#### Protein encapsulation

Quantum dots (QDs) have the capability to form complexes with proteins, including albumin, gelatin, and silk fibroin, through conjugation. The protein encapsulation process can potentially improve the biocompatibility and biodegradability of quantum dots (QDs) while offering specialized binding sites for target molecules. The regulation of quantum dot (QD) release can be achieved through protein encapsulation, utilizing the enzymatic cleavage or denaturation of protein chains^119^.

#### Silica encapsulation

Quantum dots (QDs) can be incorporated within silica shells or matrices, resulting in the formation of silica-QD composites. The process of silica encapsulation has been shown to provide a protective barrier for quantum dots (QDs), shielding them from potential oxidation or leaching. This encapsulation technique has been shown to enhance the stability and dispersibility of QDs while mitigating their inherent toxicity. The controlled release of quantum dots (QDs) can be achieved by manipulating the porosity or degradation of silica materials, allowing silica encapsulation to serve as an effective method^120^.

### Conjugation

Quantum dots (QDs) can form bioconjugates through conjugation with biomolecules, including antibodies, peptides, aptamers, and small molecules. Bioconjugates possess the ability to identify and adhere to particular receptors or antigens located on the external membrane of target cells or tissues. The liberation of quantum dots (QDs) can be initiated through diverse methods, including receptor-mediated endocytosis, protease or nuclease cleavage, or ligand-driven competitive displacement^121^. Several examples of conjugation procedures are described below.

#### Antibody conjugation

Quantum dots (QDs) can form covalent bonds with antibodies, a type of protein that exhibits a potent and specific binding affinity toward particular antigens. The utilization of antibody-conjugated quantum dots (QDs) has been demonstrated in many applications, such as immunofluorescence imaging, immunoassays, and targeted drug administration (Papyrus Bio, n.d.). Quantum dots (QDs) have been employed in conjunction with anti-human IgG antibodies for cell imaging, targeting, and fluorometric assays^122^.

#### Peptide conjugation

Quantum dots (QDs) can form covalent or noncovalent bonds with peptides composed of short amino acid sequences and interact with a diverse range of receptors or enzymes. The use of peptide-conjugated quantum dots (QDs) has demonstrated potential in several applications, such as molecular recognition, cellular uptake, and medication administration. An instance of utilizing quantum dots (QDs) linked to a peptide with an affinity for tumor cells was observed in the context of in vivo tumor imaging and detection^123,124^.

#### Aptamer conjugation

Quantum dots (QDs) can form covalent or noncovalent bonds with aptamers, nucleic acids made in a laboratory that can bind strongly and selectively to particular targets. A potential use of aptamer-conjugated quantum dots (QDs) lies in biosensing, bioimaging, and targeted treatment. Fluorescence-based thrombin detection has been seen to use quantum dots (QDs) that are linked to an aptamer that can recognize thrombin^125,126^.

#### Small-molecule conjugation

Small compounds such as medications, hormones, vitamins, or neurotransmitters can be covalently or noncovalently bonded to QDs. The potential of small-molecule-conjugated quantum dots (QDs) has been demonstrated in several applications, such as sensing, imaging, and medicinal administration. For example, quantum dots (QDs) have been coupled with folic acid for targeting and imaging folate receptor-positive cancer cells^127^.

## Toxicity and biocompatibility study

One of the unfavorable features of QDs is their large size, which can interfere with biological systems^128^. This interference can be particularly problematic in allergies, where bivalent allergen binding is required for the allergic reaction to occur^129^. Therefore, it is vital to consider the potential adverse effects of QDs on the immune system and to evaluate their immunotoxicity^128^. In this context, QDs can induce cytotoxicity via numerous mechanisms, such as necrosis, apoptosis, and autophagy. For example, they have different toxicity levels depending on the fabrication material, device size, administered dosage, administration modality, and capping substance^130^.

CdSe (cadmium selenide) is the most common material for manufacturing QDs and has significant toxicity when it accumulates in the spleen without degrading. Local neutrophil inflammation was also reported in the lungs after exposure to cadmium-based QDs. Oxidative reactions of cadmium QDs may result in reduced cadmium, leading to cell death. One of the causes of cell death is the generation of singlet oxygen species generated by the oxidation of the proteins and lipids of cells^131^.

Sometimes, endosomes and lysosomes induce cell damage by liberating heavy metals from quantum dots. Inhaled QDs have been reported to be toxic because of their internalization by endocytosis and their ability to remain in cells for a considerable time^132^. A number of severe toxicity effects are observed, such as pericardial edema, ocular edema, and spinal curvature^133,134^. Stan et al. examined the impact of Si/SiO_2_ QDs on human lung fibroblasts. According to the authors, Si/SiO_2_ QDs caused cytotoxicity by disrupting cellular homeostasis and increasing malondialdehyde (MDA) and ROS levels^135^. In addition, Si/SiO_2_ QDs disrupted actin filaments and the membrane of MRC-5 cells. Extracellular matrix turnover was also unbalanced due to a reduction in matrix metalloproteinase (MMP)-1, MMP-2, and MMP-9 activity, indicating the possibility that MMPs are risk factors for pulmonary fibrosis since SiO_2_ is one of the most commonly encountered dangerous silica agents closely associated with silicosis^136^.

A study using primary hepatocytes as a liver model found that CdSe quantum dots resulted in acute toxicity. It was found that hepatic toxicity results from the Cd selenide core attached to sulfhydryl residues of mitochondrial proteins. It has been demonstrated that the surface oxidation of quantum dots generates a reduced form of Cd that can be liberated from quantum dots and induce apoptosis. Another factor in toxicity is the core of the CdSe quantum dots. For this reason, coating the shell of QDs with metal has been demonstrated to decrease the dissolution of the core component in cells and lessen metal-induced toxicity. Despite the intrinsic toxicity of many quantum dots, group III-IV semiconductor QDs possess considerably less toxicity, making them ideal for use in biosensors and optical probes. Metal-free compounds or carbon compounds are preferred for the fabrication of QDs to reduce the potential toxicity and promote their safe application. Biocompatible functionalization is also employed to minimize the potential toxicity by incorporating the mercapto group. For example, a phospholipid micelle can also be used to encapsulate quantum dots for pharmacological applications, preserving their optical properties without altering the surface of the particles, which provides highly accurate labeling of immunological systems using QDs^137–139^.

## Immunotoxicity of QDs

**Fig. 5:**
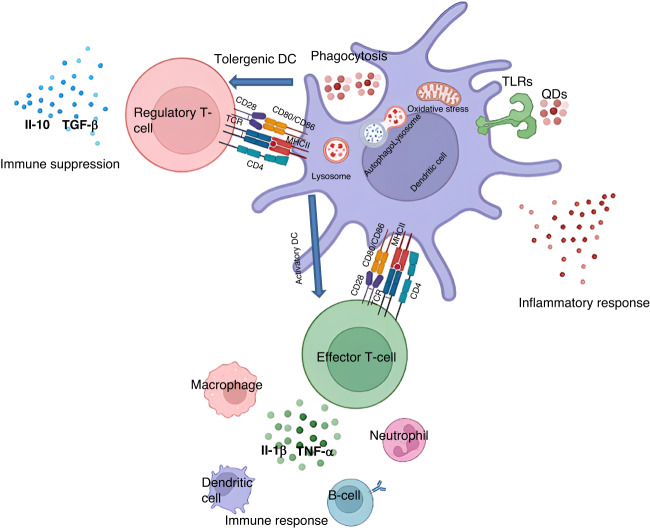
The disturbance in the immune response caused by a QD. APCs and QDs can interact, reflecting the QDs’ effects on the innate immune system by detecting surface markers and cytokines. QDs interfere with the maturation of DCs by regulating their differentiation into activator DCs or tolerogenic DCs. Furthermore, subgroups of T cells and cytokines may be detected because of the immune response induced by QDs. The image was illustrated using BioRender.com. TLR Toll-like receptor, QD quantum dot, TNFα tumor necrosis factor, TCR T-cell receptor, MHC major histocompatibility complex, TGFβ transforming growth factor β

The advancement of nanotechnology has led to significant interest in the immune compatibility of QDs, which were initially developed for medical and pharmaceutical applications. The immune system can be triggered by QD breakdown when passing through the gastrointestinal tract^140^ or deposition in the mucosal epithelium^141^. Thus, it is essential to examine how QDs interact with the immune system and evaluate the immune response to apply this technology safely (Fig. 5).

The recognition and uptake of QDs by immune cells, specifically antigen-presenting cells (APCs), are important processes. Cell uptake, cytokine production, and antigen presentation are facilitated by immune cells that initiate the immune response. QDs cannot be uptake by lymphocytes but can be uptake by macrophages and monocytes, although they are more susceptible to QDs than other human cells^142,143^. It has been reported that graphene-based QDs (GQDs) inhibit the activation of T helper type 1 (Th1) and Th17 cells; they also inhibit inflammatory responses and induce Tregs, resulting in the inhibition of inflammatory responses. Another study reported that graphene-based nanostructures promote cell growth and enhance inflammatory cell viability^144^. Zhang et al. (2011) observed that CD86 and CD80 decreased in porcine monocyte-derived DCs treated with QD-655^142^. It was inferred that a suppressive effect on DC maturation was likely responsible for this result. In line with this hypothesis, it was found that immature DCs (iDCs) possess a low level of surface costimulatory molecules (such as B7) and have low immunogenicity, resulting in DCs serving as tolerogenic agents^145–147^.

QDs disrupted RAW264.7’s ability to phagocytose^148^; for example, GQDs could stimulate macrophage polarization toward the alternative anti-inflammatory type (the M2 type) while suppressing the classic pro-inflammatory type (the M1) polarization, leading to the upregulation of Treg cells. Additionally, CdTe-QDs and Ag_2_Se-QDs can polarize microglia into the M1 phenotype and trigger an inflammatory response by activating NLRP3 through TLR/MyD88^149^. ZnS modification decreased the inflammation triggered by Cd-QDs 154, while other QDs could induce neutrophil autophagy 155, which in turn caused inflammation^150^.

Other researchers reported immunotoxic effects of QDs inducing cytokine production and secretion. CdSe/ZnS QDs covered in COOH triggered a more significant inflammatory response than other QDs^151^. A low concentration of GQD treatment increased the expression levels of IL-8, IL-1β, and TNF-α in macrophages; however, the opposite effect was observed when a high number of GQDs was applied^152^. Another study showed that GQD treatment decreased splenic Th1 cells and decreased IL-12 production in colitis mice^153^. This study found an increase in Tregs and IL-10 and TGF-1 159 expression levels. GQD treatment also increased IL-10 levels in DCs, whereas IL-23, IL-27, and IL-12 levels declined in DCs^154^.

QDs may also affect the complement system by adhering to complement proteins, although QDs coated with lipids exhibited only a modest affinity to plasma proteins^155,156^. GQDs also triggered the alternative pathway initiated by C3b, resulting in a higher level of seven complement components implicated in forming a membrane attack complex (MAC)^157^. The literature on this topic indicates that complement activation may trigger an inflammatory response.

The majority of QDs enter lysosomes after cell internalization. For this reason, autophagy is an essential mechanism underlying QD toxicity due to degradation by lysosomes. A number of QDs have been shown to induce autophagy according to their size^152,158^. However, QDs aggregated into microparticles have not been shown to trigger autophagy^159^. One study reported that GQDs stimulate autophagy more often than apoptosis, possibly indicating that macrophages may employ autophagy as a defensive response to GQDs^152^.

In contrast, a large GQD (40 nm) inhibited autophagy, alleviating concanavalin A (ConA)-induced liver damage^160^. Moreover, Fan et al. reported that inhibiting autophagy prevented nanotoxicity caused by Cd-based QDs^161^. Despite recent progress, more research is needed to fully understand the function of QD-mediated autophagy in regulating the immune system.

QDs can also interfere with inflammatory responses that change the immune system’s function^162^. For example, CdTe-QDs markedly raised the counts of white blood cells (WBCs) and caused a significant increase in acute phase proteins (serum amyloid A, SAA) at high doses^163^. It has been reported that immune cells that phagocytose QDs move to the immune tissue, causing inflammation in those tissues instead of systemic inflammation^148^. For example, GQDs inhibit Th1/Th17 differentiation, enhance M1 to M2 differentiation, and promote Treg infiltration^153^. Accordingly, the induction of tolerogenic DCs through autophagy by GQDs inhibited Th1 pro-inflammatory development^154^. Thus, QDs profoundly influence autophagy, which is crucial to immune-mediated inflammatory functions. The use of these materials in allergy research can be enhanced by gaining more profound knowledge of how QDs modulate autophagy.

QDs can also cause an unbalanced redox state in cells, which may result in increased production of ROS and the generation of pro-inflammatory cytokines. The Ag_2_Se-QDs increased inflammatory reactions in microglia by enhancing ROS production and activating the NLRP3 inflammasome^149^. A recent in vitro study revealed that GQDs restricted macrophage activation (J774 cell line) and T-cell activation (Jurkat) and reduced free radical nitric oxide (NO) release in macrophages^160^. Furthermore, GQDs diminish ROS levels and initiate autophagy, enhancing tolerogenic DC differentiation and blocking the polarization of Th1 cells in the presence of ROS^154^. More studies are needed to clarify the links between QD-mediated autophagy, oxidative stress, and inflammation. Therefore, it is important to carefully design and evaluate quantum dots for biocompatibility and immunotoxicity before using them for biomedical purposes. The following section reviews strategies to overcome the toxicity of quantum dots.

## Strategies to minimize QD toxicity

Quantum dots have attracted considerable interest regarding biomedical applications due to their distinctive optical characteristics and potential for transformative advancements in various domains, including imaging, drug transport, gene therapy, and immunoassays. Nevertheless, the toxicity associated with conventional cadmium-based quantum dots (QDs) has led to the investigation of several approaches aimed at reducing their detrimental impact^134^. This concern stems from the release of heavy metal ions and the consequences resulting from their nanoscale dimensions, prompting the development of methods to alleviate these issues. In this context, new developments in nontoxic quantum dots (QDs) and their applications in the field of biomedicine present encouraging prospects for tackling these issues^164^.

One of the primary obstacles encountered in using quantum dots (QDs) for medicinal purposes is their inherent toxicity. One approach to mitigate the toxicity of quantum dots (QDs) entails the alteration of their core-shell architecture. Using conventional semiconductor quantum dots (QDs) composed of heavy metal ions, such as those based on cadmium, raises concerns regarding their possible adverse effects on the environment and human health^165^. Core-shell modifications refer to a process in which a core quantum dot (QD) is enclosed within a shell material compatible with biological systems. This shell material physically prevents the release of heavy metal ions from the core, hence improving the stability of the core-shell structure. This methodology is demonstrated by implementing polymeric shell coatings or water-soluble ligands to passivate the core QDs^133^. The alterations mentioned above reduce the emission of harmful ions and offer the potential for bioconjugation, facilitating precise targeting and regulated administration inside biological contexts. One approach involves the encapsulation of individual quantum dot nanocrystals within a phospholipid block-copolymer micelle, as described in a previous study^166^. The micelles under consideration possess a hydrophobic core capable of accommodating individual CdSe QDs coated with a ZnS layer. These micelles are characterized by their lipid composition and the presence of hydrophilic polymer branches. The modified QDs demonstrated a low level of toxicity, with a concentration of less than 5 × 10^9^ nanocrystals per cell. Additionally, they exhibited high stability and minimal photobleaching effects upon injection into Xenopus embryos^166^.

One potential approach to mitigate the toxicity of QDs is the advancement of formulations devoid of heavy metals and metals. These formulations employ nontoxic and environmentally acceptable starting materials, avoiding the possible risks associated with heavy metal-based quantum dots. Ternary group I-III-VI QDs exemplify heavy metal-free QDs. These QDs consist of elements from group I (Cu, Ag), group III (Al, Ga, In, Tl), and group VI (S, Se, Te). They provide a safer alternative option with adjustable optical characteristics. Certain QDs are classified in the I-III-VI group, including AgInS_2_, CuInS_2_, and ZnS–AgInS_2_. QDs exhibit enhanced manipulation of the energy gap between the valence and conduction bands^167^. Alterations in their size and composition can modify the optical properties of these materials. As an example, CuInS_2_ possesses a narrow energy band gap of 1.45 eV, resulting in its emission inside the near-infrared (NIR) region^168^. The emission peak of CuInS_2_ may be altered from 693 to 835 nm by manipulating the reaction temperature, which can be attributed to the quantum size effect^168^. Furthermore, CuInS_2_ QDs exhibit characteristic behavior associated with quantum confinement and display a broad peak in the more extended wavelength region. Using a ZnS inorganic shell has been found to enhance the quantum yield and emission lifetimes of QDs while concurrently mitigating the release of Cd_2_^+^ ions and safeguarding QDs against oxidation^167–169^.

Carbon quantum dots (CQDs) have emerged as a potential solution to mitigate the toxicity associated with QDs in biomedical applications. Quasispherical carbon nanoparticles, commonly called CQDs, are smaller than 10 nm and are generated from several carbon-rich sources^167,170^. In contrast to conventional QDs that contain cadmium and lead, CQDs consist of carbon-rich precursors. This composition mitigates the potential hazards of heavy metal contamination and the resulting toxicity^101^. The lack of heavy metals in CQDs alleviates concerns regarding the release of harmful ions and their subsequent buildup in biological systems, making CQDs a comparatively safe choice for biomedical applications^171^.

Additionally, CQDs have distinctive photoluminescent characteristics that can be adjusted by manipulating their size, surface modifications, and fabrication techniques. The tunable optical features of CQDs allow fluorescence emission within a defined wavelength range, encompassing both the visible and near-infrared regions. These properties render them highly suitable candidates for bioimaging applications since they can serve as contrast agents for visualizing cellular structures and processes^171^. Moreover, CQDs have demonstrated exceptional biocompatibility in numerous in vitro investigations^172^. In contrast to cadmium-based QDs, which have been shown to induce oxidative stress and DNA damage, CQDs exhibit little cytotoxicity toward cellular systems. The distinctive electrical and chemical characteristics of CQDs enable their utilization as carriers for medication delivery, imaging agents, and biomolecular sensors while maintaining cellular viability^173^. Moreover, the relatively small size of CQDs facilitates effective cellular internalization, making them suitable for precise imaging and therapeutic purposes. Furthermore, CQDs exhibit potential in the field of medicinal applications in addition to their imaging capabilities. The suitability of nanoparticles as drug delivery systems is attributed to their biocompatibility and capacity to transport payloads, such as medicines or biomolecules. CQDs can undergo functionalization to effectively augment drug encapsulation, enable regulated release mechanisms, and facilitate targeted delivery to specific tissues or cells^171,173^. The abovementioned capability presents opportunities for advancing customized medicine and implementing more efficacious treatment approaches. Consequently, CQDs exhibit a wide range of properties that make them adaptable for many biomedical applications.

Using nucleotides and amino acids as precursors for QD synthesis is a prominent strategy in current trends. This approach highlights the promising capabilities of biomolecule-derived QDs in imaging and treatment applications. The functional groups (R groups) present in the side chains of natural amino acids make them suitable as precursors for the programmed synthesis of carbon dots produced from biomolecules, commonly referred to as biodots, possessing specific desirable qualities^174^. The study by Zheng et al. represents the initial comprehensive investigation into the principles governing the design of materials for biodot synthesis^175^. The authors employ a green hydrothermal method to analyze the synthesis process utilizing 20 naturally occurring α-amino acids. Comprehensive characterization is used to establish the structure-property relationship between the amino acid precursors and the photoluminescent properties of the resulting amino acid biodots, commonly referred to as AA dots. The authors demonstrate that the AA dots exhibit remarkable biocompatibility and exceptional intracellular uptake, making them highly appealing for imaging applications^176^. Zheng et al. (2019) also documented the utilization of nucleotide-derived biodots for imaging and therapeutic purposes^175^. The four fundamental nucleotides of DNA are employed as precursor materials in the synthesis of fluorescent nucleotide biodots (N-dots) by a one-pot hydrothermal synthesis method. In this study, a variety of N-dots were synthesized and characterized. Notably, adenosine triphosphate (ATP) dots exhibited the highest fluorescence quantum yield (QY), with a value of 13.9%^177^.

Moreover, adenosine diphosphate (ADP)-dots show exceptional photostability, retaining 97.6% of their photoluminescence intensity even after continuous UV illumination for 30 min^175^. DNA biodots exhibit remarkable durability when exposed to UV irradiation and DNase enzymatic activity^178^. In addition, these particles possess distinctive physiochemical characteristics, including their remarkably small dimensions that facilitate cellular uptake, adjustable photoluminescence properties that enable effective bioimaging, exceptional solubility in aqueous environments, notable chemical and photostability, and remarkable singlet oxygen quantum yield, which is accompanied by inherent biocompatibility for applications in photodynamic therapy^179^. These qualities hold significant importance in the context of theranostic applications.

The investigation into nontoxic QDs and associated methodologies provides encouraging avenues for mitigating the toxicity of QDs in biomedical applications. Additional investigation is required to substantiate the safety and effectiveness of these approaches in diverse biological scenarios. Furthermore, establishing precise detection technologies and systematic approaches for toxicity evaluation is vital to guarantee the biocompatibility of QDs. Incorporating in situ, real-time, and expeditious quantitative analytic techniques will facilitate advancements in comprehending the behavior of QDs within biological systems.

The pursuit of mitigating the toxicity of quantum dots in biomedical applications has prompted the emergence of novel approaches. The implementation of core-shell modifications, heavy metal-free and metal-free QD formulations, silicon quantum dots, and thorough toxicity assessments are facilitating the development of safer and more efficient utilization of QDs in diverse biological applications. As scientific progress continues, incorporating these approaches will play an important role in harnessing the full potential of QDs in transforming various domains, including imaging, drug administration, and therapeutic interventions, all while mitigating their adverse effects on human health and the ecosystem.

## Challenges and prospects

Quantum dots (QDs) have emerged as a revolutionary technology that can potentially significantly transform the field of allergic illness detection and treatment. These entities’ distinctive optical and physicochemical characteristics present intriguing possibilities for precise diagnostic techniques, focused therapeutic approaches, and novel immune-modulation methods. However, the fulfillment of these commitments will involve the navigation of a multifaceted array of difficulties, which encompass issues regarding the compatibility of biological systems, intricacies associated with precise targeting, obstacles related to regulatory approval, the difficulty of translating research findings into practical applications, and the intricate interplay between quantum qualities and the compatibility of biological systems^50^.

### Biocompatibility

A substantial obstacle to the possible incorporation of QDs into allergic disease management is the challenge of ensuring biocompatibility. Traditional QDs, typically fabricated using heavy metals such as cadmium, possess exceptional optical characteristics that make them attractive candidates for biomedical purposes^165^. However, the release of heavy metal ions is a drawback that raises concerns about their suitability for in vivo applications. The complex interaction between QDs and the sophisticated immune system introduces additional layers of intricacy to this particular difficulty. The complex interplay between QDs and the immune system requires a comprehensive understanding of the possible immunomodulatory effects of QDs and their potential implications for enduring immunological reactions^132^.

Quantum dots (QDs) exhibit a unique ability to engage with immune cells, functioning as practical tools for cell-specific targeting, detection, and therapy. Nevertheless, there are concerns over the possible immunotoxic effects these interactions may induce. The potential release of heavy metal ions from conventional QDs can elicit immunological responses, which may have detrimental effects on the homeostasis of the immune system. The crux of the matter lies in comprehending the complex network of interactions between QDs and immune cells and the need to determine whether these interactions can shift the equilibrium toward unfavorable immune modulation or persistent immunological dysregulation^148^.

The successful resolution of the biocompatibility challenge necessitates interdisciplinary collaboration among professionals in materials science, immunology, and clinical practice. In this context, biocompatible coatings and new materials can be applied to mitigate the issue of heavy metal-related toxicity while preserving the optical properties of QDs. A thorough investigation of the compatibility between QDs and the complex mechanisms of immune responses will require meticulous and comprehensive in vitro and in vivo experiments. Attaining genuine biocompatibility requires not only the prevention of immediate hazardous reactions but also the assurance that QDs can easily integrate with the immunological environment without causing persistent immune changes. Therefore, the exploration of integrating QDs into the management of allergic diseases will involve a comprehensive examination of their biocompatibility characteristics. This entails matching their exceptional optical properties with the complex immunological environment to provide safer and more efficient interventions.

### Precision targeting and immunomodulation

Quantum dots (QDs) present an intriguing possibility in allergic disease management, as they can precisely target immune cells that are specific to allergens. This could pave the way for innovative therapeutic strategies. The fulfillment of this commitment relies on coordinating a multitude of intricate factors, including the size of QDs, their surface chemistry, and the intricate interactions between QDs and immune cell receptors, which must harmoniously align.

The task at hand involves identifying a precise balance between effective targeting and the reduction of unintended consequences, which necessitates a comprehensive comprehension of immunobiology. Although the potential benefits of utilizing QD technology for precision targeting are apparent, it is essential to acknowledge the complexity of the intercellular environment. Care must be taken to avoid missteps that could induce unwanted immunological responses. Success is contingent upon the molecular manipulation of quantum dots (QDs) to optimize their compatibility with the receptor composition of immune cells that are specific to allergens. This would allow the QDs to selectively bind and regulate immune responses in a precise and regulated manner.

Accurate targeting will necessitate the careful development and construction of QDs, customizing their physical and chemical properties to align with the intricate receptors found on immune cells. Surface functionalization techniques, such as the attachment of ligands that exhibit compatibility with immune receptors, will facilitate the operation of QDs as molecular keys, enabling the selective activation of particular subsets of immune cells^151^. Successful molecular orchestration necessitates a comprehensive understanding of the intricate immunological responses characteristic of allergic disorders because the immune landscape and allergens might vary significantly among individuals.

The objective of precision targeting is not only to reduce unintended effects but also to enhance therapeutic approaches. Through the modulation of immune cell responses at the molecular level, QDs can recalibrate immunological reactions, thereby mitigating the excessive reactivity commonly observed in allergic disorders. Attaining such a high degree of accuracy will require a collective effort, including experts in immunology, materials science, and clinical practice, to unravel the complex interplay between QDs and immune cells. The progress of science suggests that QDs could potentially transform allergic disease management. However, this promise relies on the ability to achieve precise targeting without unwanted disruption to the intricate immune system.

### Regulatory approval and clinical translation

The successful integration of QDs as a valuable instrument for treating allergic diseases is contingent upon obtaining regulatory approval and facilitating clinical translation. The transition from innovative scientific investigations to practical applications in clinical settings requires a persistent commitment to thorough validation, encompassing safety assessments, effectiveness, and consistent outcomes.

Regulatory bodies enforce rigorous requirements for assessing the feasibility of therapies based on QDs. These standards establish a comprehensive framework that requires QDs to meet rigorous criteria before proceeding to clinical trials and eventually becoming part of routine medical practice. Researchers, manufacturers, and physicians are responsible for effectively bridging the gap between creative advancements and regulatory requirements. Comprehensive preclinical investigations to elucidate the complex interactions between QDs and the immune system are of utmost importance. These studies must provide an in-depth understanding of quantum dot (QD) behavior, their possible toxicity, and their long-term effects, thereby establishing the basis for safety assessments.

It is equally crucial to interact proactively with regulatory organizations. The establishment of open communication channels, collaborative discussions, and public dissemination of research findings play a pivotal role in facilitating regulators’ understanding of the potential of quantum dots (QDs) and providing guidelines regarding acceptable risk and safety thresholds.

The successful translation of clinical research relies heavily on a rigorous validation procedure encompassing every stage from initial laboratory experiments to the final implementation at the patient’s bedside. This entails showcasing consistent performance across multiple groups of patients, clarifying the impact of quantum dots on immunological responses, and uncovering any unexpected complications.

In essence, obtaining regulatory clearances and conducting clinical translation is a transformative process wherein the promise of QDs materializes into concrete advantages for individuals contending with allergic disorders. This process relies on the dedication of researchers, physicians, and regulatory authorities to navigate the complex terrain of innovation, safety, and patient-centered care.

### Translational challenges and quantum properties

As the potential of QDs becomes increasingly viable for practical clinical use, a unique array of obstacles arises in translating this technology into real-world applications. Transitioning from controlled laboratory settings to intricate clinical situations necessitates the scalability, reproducibility, and standardization of QD synthesis and functionalization procedures^31^. These variables are crucial in guaranteeing consistent and dependable performance across a wide range of applications and cohorts of patients. However, the complexity of this task increases as the extraordinary quantum features that distinguish quantum dots become involved. The fluorescence characteristics of QDs, attributed to the quantum confinement phenomenon, are remarkable and provide a wide range of colors for imaging and therapeutic applications^33^. The preservation of these exceptional characteristics while ensuring biocompatibility is crucial and is not easily accomplished.

The maintenance of this intricate balance necessitates a nuanced interplay among various components. The ability to accurately manipulate QD size is of utmost importance since it directly impacts their optical properties and potential interactions with immune cells. To facilitate the seamless navigation of QDs within the biological environment, it is imperative to carefully control the intricacies of surface chemistry. Rigorous management is necessary to prevent the inadvertent activation of immune responses. Moreover, the potential threat posed by heavy metal toxicity is a significant concern, necessitating the development of effective measures to prevent the release of hazardous ions.

The convergence of these problems highlights the complex nature of the current undertaking. To effectively integrate quantum dots (QDs) into clinical practice, researchers, materials scientists, and clinicians must establish collaborative and cohesive partnerships. This collaboration necessitates the harmonious integration of scientific expertise, technological advancements, and clinical knowledge to overcome the challenges associated with translating QD research from the controlled environment of laboratories to the complex and dynamic realm of real-world clinical settings.

### Innovative solutions and collaborative endeavors

As the complexity of using QDs in managing allergic diseases becomes increasingly apparent, joint efforts in pioneering solutions emerge as a prominent guiding force. The convergence of biocompatibility and the conservation of optical properties prompts the development of creative tactics that drive advancements in the field.

The development of biocompatible coatings and the emergence of heavy metal-free formulations represent promising advancements in the field. These approaches utilize advanced material science techniques to develop QDs that retain their remarkable optical characteristics and avoid the drawbacks associated with the toxicity of heavy metals. These novel methods provide QDs that can effectively be navigating the complex biological environment. Such QDs could serve as precise tools in therapies for allergic diseases.

Surface engineering techniques play a crucial part in the above developments. Modifications to their surface can transform QDs into molecular entities that can activate particular subsets of immune cells^31,33^. Customized QDs can be designed to finely regulate immune responses, thereby mitigating the excessive reactivity that serves as the foundation for allergic conditions.

Effectively utilizing these solutions is intricately linked to the ethos of cooperation. The crucial nature of interdisciplinary collaboration among materials scientists, immunologists, physicians, and regulatory agencies cannot be overstated. Every field of expertise makes a distinct contribution to the overall composition. Materials scientists exhibit exceptional skill in creating QDs; immunologists unravel the complexities of the immune system; physicians offer valuable practical insights; and regulators guarantee compliance with rigorous standards.

The collaborative endeavor, driven by a collective vision and common problems, is the environment in which theoretical concepts transform into practical and influential implementations. The collaborative nature of this attitude overcomes limitations. It cultivates a harmonious interaction of concepts, creating avenues that negotiate complex obstacles and steer quantum dots toward their potential contribution to mitigating allergic disorders. The revolutionary potential of quantum dots in allergic disease management could become evident through implementing new solutions and collaborative efforts. These advancements illuminate a promising route forward.

### Concluding reflections

Integrating QDs into allergic illness detection and therapy represents a convergence of immense possibilities and complex obstacles. When faced with the task of navigating unfamiliar territory, significant obstacles must be overcome. These issues require careful consideration and the development of creative approaches to be successfully addressed. However, the unquestionable potential of QDs in revolutionizing the management of allergic diseases presents an enticing prospect, offering a transformative outlook for the future.

The resolution of biocompatibility challenges is essential for the successful translation of this technology into clinical applications. The field of QDs has a wide range of remarkable optical characteristics. However, successfully integrating these qualities into practical medical applications relies on their compatibility with biological systems. Researchers navigate the intricate balance between optical properties and patient well-being by developing biocompatible coatings and adopting formulas free from heavy metals.

The complexities of immunomodulation present an additional crucial frontier. The orchestration of immune responses necessitates meticulous precision since quantum dots (QDs) interact with immune cells, presenting the possibility of affecting abnormal reactions that serve as the foundation for allergic disorders. Researchers seek to understand and control the intricate and synergistic interplay of quantum dot (QD) size, surface chemistry, and immune cell receptors.

Achieving clinical integration involves navigating through regulatory requirements and overcoming translational obstacles. This necessitates thorough validation and a smooth transition of research findings from laboratory settings to practical applications in the real world. The collaborative tableau, with researchers, clinicians, and regulatory organizations working together, enhances the depth of this process by integrating many perspectives and providing guidance to translate quantum dots (QDs) into a tangible clinical reality^31,33^.

In conclusion, the potential of QDs is currently at a critical point where scientific creativity, interdisciplinary cooperation, and patient-focused advancements intersect. The prospect of individualized diagnoses and therapies for allergic diseases is encouraging. By carefully manipulating the complex interaction between quantum characteristics and biocompatibility, scientists and healthcare practitioners are on the verge of revolutionizing allergic illness management. The potential transformation of patient outcomes, formerly constrained by various limits, holds the promise of becoming a paradigm shift in medicine, opening up new avenues for advancements and possibilities. The development of quantum dots represents a promising avenue for advancing allergic disease management. This achievement results from creativity, dedication, and interdisciplinary collaboration that have enabled the exploration of novel applications of this technology.

## Conclusion

QDs offer innovative solutions and are an alternative technology that can enhance therapy and diagnosis for allergies. This is due to their unique properties, including easy tunability, making them a versatile diagnostic tool. A wide range of QD structures are available, and controlling their physicochemical properties may enable the development of more efficient compounds to achieve therapeutic and diagnostic goals. For example, QDs can (a) increase the number of antigens that target specific IgE antibodies, (b) improve signal detection in experiments, and (c) detect allergen concentrations in trace amounts.

A fundamental prerequisite for successfully translating QD platforms into the clinical setting is the ability to provide evidence of efficacy, safety, and therapeutic superiority over currently available allergy treatments (e.g., asthma), along with cost-effective product manufacturing.

Therefore, future research should focus on designing QDs that are more biocompatible and have specific biochemical and optical characteristics to trigger a desirable response. In vitro diagnosis and detection of food allergens appear to be the most promising applications that may soon be universally adopted.

## References

[1] Stern, J., Pier, J. & Litonjua, A. A. In *Semin Immunopathol*. 5–15 (Springer, 2020). 10.1007/s00281-020-00785-1 32020334

[2] Ogulur I (2021). Advances and highlights in biomarkers of allergic diseases. Allergy.

[3] Gholami A, Mohammadi F, Ghasemi Y, Omidifar N, Ebrahiminezhad A (2020). Antibacterial activity of SPIONs versus ferrous and ferric ions under aerobic and anaerobic conditions: a preliminary mechanism study. IET Nanobiotechnol..

[4] Raee MJ, Ebrahiminezhad A, Gholami A, Ghoshoon MB, Ghasemi Y (2018). Magnetic immobilization of recombinant E. coli producing extracellular asparaginase: an effective way to intensify downstream process. Sep. Sci. Technol..

[5] Gholami A (2016). Magnetic properties and antimicrobial effect of amino and lipoamino acid coated iron oxide nanoparticles. Minerva Biotecnol..

[6] Ikumapayi O, Akinlabi E, Adeoye A, Fatoba S (2021). Microfabrication and nanotechnology in manufacturing system–An overview. Mater. Today-Proc..

[7] Asadi K, Gholami A (2021). Virosome-based nanovaccines; a promising bioinspiration and biomimetic approach for preventing viral diseases: A review. Int. J. Biol. Macromolecules.

[8] Ekimov AI, Onushchenko AA (1981). Quantum size effect in three-dimensional microscopic semiconductor crystals. JETP Lett..

[9] Rocha TL, Mestre NC, Sabóia-Morais SMT, Bebianno MJ (2017). Environmental behaviour and ecotoxicity of quantum dots at various trophic levels: a review. Environ. Int.

[10] Ventola CL (2017). Progress in nanomedicine: approved and investigational nanodrugs. PT.

[11] Mousavi SM (2022). Bioactive graphene quantum dots based polymer composite for biomedical applications. Polymers.

[12] Mousavi SM (2021). Precise blood glucose sensing by nitrogen-doped graphene quantum dots for tight control of diabetes. J. Sens..

[13] Paradise J (2019). Regulating nanomedicine at the food and drug administration. AMA J. Ethics.

[14] Li S (2020). Targeted tumour theranostics in mice via carbon quantum dots structurally mimicking large amino acids. Nat. Biomed. Eng..

[15] Mayorga C, Perez‐Inestrosa E, Rojo J, Ferrer M, Montañez MI (2021). Role of nanostructures in allergy: Diagnostics, treatments and safety. Allergy.

[16] Male KB, Lachance B, Hrapovic S, Sunahara G, Luong JH (2008). Assessment of cytotoxicity of quantum dots and gold nanoparticles using cell-based impedance spectroscopy. Anal. Chem..

[17] Taniguchi S, Green M, Rizvi SB, Seifalian A (2011). The one-pot synthesis of core/shell/shell CdTe/CdSe/ZnSe quantum dots in aqueous media for in vivo deep tissue imaging. J. Mater. Chem..

[18] Reshma V, Mohanan P (2019). Quantum dots: applications and safety consequences. J. Lumin.

[19] Mousavi SM (2022). The pivotal role of quantum dots-based biomarkers integrated with ultra-sensitive probes for multiplex detection of human viral infections. Pharmaceuticals.

[20] Idowu M, Lamprecht E, Nyokong T (2008). Interaction of water-soluble thiol capped CdTe quantum dots and bovine serum albumin. J. Photochem Photobio. A Chem..

[21] Gholami A, Mokhtary M, Nikpassand M (2020). Glycolic acid‐supported cobalt ferrite‐catalyzed one‐pot synthesis of pyrimido [4, 5‐b] quinoline and indenopyrido [2, 3‐d] pyrimidine derivatives. Appl. Organomet. Chem..

[22] Mousavi, S. M. et al. Bioinorganic synthesis of sodium polytungstate/polyoxometalate in microbial kombucha media for precise detection of doxorubicin. *Bioinorg. Chem. Appl.* **2022**, 1–12 (2022). 10.1155/2022/2265108 PMC9377961 35979186

[23] Mousavi SM (2022). Biomedical applications of an ultra-sensitive surface plasmon resonance biosensor based on smart MXene quantum dots (SMQDs). Biosensors.

[24] Wang J, Han S, Ke D, Wang R (2012). Semiconductor quantum dots surface modification for potential cancer diagnostic and therapeutic applications. J. Nanomater..

[25] Clift MJ, Stone V (2012). Quantum dots: an insight and perspective of their biological interaction and how this relates to their relevance for clinical use. Theranostics.

[26] Mousavi SM (2022). Recent Advances in Inflammatory Diagnosis with Graphene Quantum Dots Enhanced SERS Detection. Biosensors.

[27] Chern M, Kays JC, Bhuckory S, Dennis AM (2019). Sensing with photoluminescent semiconductor quantum dots. Methods Appl. Fluoresc..

[28] Shu Y (2020). Quantum dots for display applications. Angew. Chem..

[29] Wagner AM, Knipe JM, Orive G, Peppas NA (2019). Quantum dots in biomedical applications. Acta Biomaterialia.

[30] Kargozar S (2020). Quantum dots: a review from concept to clinic. Biotechnol. J..

[31] Abdellatif, A. A., Younis, M. A., Alsharidah, M., Al Rugaie, O. & Tawfeek, H. M. Biomedical applications of quantum dots: overview, challenges, and clinical potential. *Int. J. Nanomed.* **17**, 1951–1970 (2022). 10.2147/IJN.S357980 PMC9076002 35530976

[32] Su G, Liu C, Deng Z, Zhao X, Zhou X (2017). Size-dependent photoluminescence of PbS QDs embedded in silicate glasses. Optical Mater. express.

[33] Bera D, Qian L, Tseng T-K, Holloway PH (2010). Quantum dots and their multimodal applications: a review. Materials.

[34] Murray CB, Kagan CR, Bawendi MG (2000). Synthesis and characterization of monodisperse nanocrystals and close-packed nanocrystal assemblies. Annu. Rev. Mater. Sci..

[35] Pleskova, S., Mikheeva, E. & Gornostaeva, E. Using of quantum dots in biology and medicine. *Cell. Mol. Toxicol. Nanopart.* **1048**, 323–334 (2018). 10.1007/978-3-319-72041-8_19 29453547

[36] Schmidt R, Krasselt C, Göhler C, von Borczyskowski C (2014). The fluorescence intermittency for quantum dots is not power-law distributed: a luminescence intensity resolved approach. Acs Nano.

[37] Walling MA, Novak JA, Shepard JRE (2009). Quantum dots for live cell and in vivo imaging. Int. J. Mol. Sci..

[38] Samiraninezhad N, Asadi K, Rezazadeh H, Gholami A (2023). Using chitosan, hyaluronic acid, alginate, and gelatin-based smart biological hydrogels for drug delivery in oral mucosal lesions: A review. Int. J. Biol. Macromolecules.

[39] Le N, Zhang M, Kim K (2022). Quantum dots and their interaction with biological systems. Int. J. Mol. Sci..

[40] Liu Y-Y (2021). Fate of CdSe/ZnS quantum dots in cells: endocytosis, translocation and exocytosis. Colloids Surf. B: Biointerfaces.

[41] Zhang M, Kim DS, Patel R, Wu Q, Kim K (2022). Intracellular trafficking and distribution of Cd and InP quantum dots in HeLa and ML-1 thyroid cancer cells. Nanomaterials.

[42] Mo D (2017). Cadmium-containing quantum dots: properties, applications, and toxicity. Appl. Microbiol. Biotechnol..

[43] Kumar R, Kulkarni A, Nagesha DK, Sridhar S (2012). In vitro evaluation of theranostic polymeric micelles for imaging and drug delivery in cancer. Theranostics.

[44] Nguyen KC, Rippstein P, Tayabali AF, Willmore WG (2015). Mitochondrial toxicity of cadmium telluride quantum dot nanoparticles in mammalian hepatocytes. Toxicological Sci..

[45] Wang Z, Tang M (2021). The cytotoxicity of core-shell or non-shell structure quantum dots and reflection on environmental friendly: A review. Environ. Res..

[46] Chakravarthy KV (2011). Doxorubicin conjugated quantum dots to target alveolar macrophages/inflammation. Nanomedicine.

[47] Qiu J (2015). Fluorescent graphene quantum dots as traceable, pH-sensitive drug delivery systems. Int. J. Nanomed..

[48] Wang L (2022). Easy synthesis and characterization of novel carbon dots using the one-pot green method for cancer therapy. Pharmaceutics.

[49] Chilakamarthi U, Giribabu L (2017). Photodynamic therapy: past, present and future. Chem. Rec..

[50] Tabish TA (2018). Biocompatibility and toxicity of graphene quantum dots for potential application in photodynamic therapy. Nanomedicine.

[51] Algorri JF, Ochoa M, Roldán-Varona P, Rodríguez-Cobo L, López-Higuera JM (2021). Photodynamic therapy: a compendium of latest reviews. Cancers.

[52] Niculescu A-G, Grumezescu AM (2021). Photodynamic therapy—an up-to-date review. Appl. Sci..

[53] Martynenko I (2015). Chlorin e6–ZnSe/ZnS quantum dots based system as reagent for photodynamic therapy. Nanotechnology.

[54] Lu D, Tao R, Wang Z (2019). Carbon-based materials for photodynamic therapy: a mini-review. Front. Chem. Sci. Eng..

[55] Palui G, Aldeek F, Wang W, Mattoussi H (2015). Strategies for interfacing inorganic nanocrystals with biological systems based on polymer-coating. Chem. Soc. Rev..

[56] Volkov Y (2015). Quantum dots in nanomedicine: recent trends, advances and unresolved issues. Biochem. Biophys. Res. Commun..

[57] Aizik G (2019). Liposomes of quantum dots configured for passive and active delivery to tumor tissue. Nano Lett..

[58] Nasrin A, Hassan M, Gomes VG (2020). Two-photon active nucleus-targeting carbon dots: enhanced ROS generation and photodynamic therapy for oral cancer. Nanoscale.

[59] Pichler WJ (2019). Immune pathomechanism and classification of drug hypersensitivity. Allergy.

[60] Diamant Z (2019). Toward clinically applicable biomarkers for asthma: an EAACI position paper. Allergy.

[61] Ansotegui IJ (2020). IgE allergy diagnostics and other relevant tests in allergy, a World Allergy Organization position paper. World Allergy Organ J..

[62] Mayorga C (2016). In vitro tests for drug hypersensitivity reactions: an ENDA/EAACI Drug Allergy Interest Group position paper. Allergy.

[63] Peña-Mendizabal E (2022). Synthesis of skeletally diverse β-lactam haptens for the in vitro diagnosis of IgE-mediated drug allergy. Chem. Commun..

[64] Brockow K (2021). Detection of drug-specific immunoglobulin E (IgE) and acute mediator release for the diagnosis of immediate drug hypersensitivity reactions. J. Immunol. Methods.

[65] Hoffmann H (2015). The clinical utility of basophil activation testing in diagnosis and monitoring of allergic disease. Allergy.

[66] Mayorga, C., Fernández, T. D., Fernandez-Santamaría, R. & Rodriguez-Nogales, A. in *Cutaneous Drug Hypersensitivity.* 91–97 (Springer, 2022).

[67] Hamilton RG, Adkinson NF (2004). In vitro assays for the diagnosis of IgE-mediated disorders. J. Allergy Clin. Immunol..

[68] Matricardi P, Kleine-Tebbe J (2016). Molecular allergology between precision medicine and the choosing wisely initiative. Clin. Exp. Allergy.

[69] Bojcukova J, Vlas T, Forstenlechner P, Panzner P (2019). Comparison of two multiplex arrays in the diagnostics of allergy. Clin. Transl. Allergy.

[70] Yan-Bing T (2022). Electrochemical sensor for the detection of eosinophil cationic protein as a marker of allergic rhinitis based on colloidal quantum dots. Chin. J. Anal. Chem..

[71] Shen B (2020). Clinical evaluation of a rapid colloidal gold immunochromatography assay for SARS-Cov-2 IgM/IgG. Am. J. Transl. Res.

[72] Doña I, Torres MJ, Montañez MI, Fernández TD (2017). In vitro diagnostic testing for antibiotic allergy. Allergy Asthma Immunol. Res.

[73] Mayorga C, Perez-Inestrosa E, Molina N, Montañez MI (2016). Development of nanostructures in the diagnosis of drug hypersensitivity reactions. Curr. Opin. Allergy Clin. Immunol..

[74] Berlina AN (2013). Quantum-dot-based immunochromatographic assay for total IgE in human serum. PLoS ONE.

[75] Medina‐Sánchez M, Miserere S, Cadevall M, Merkoçi A (2016). Enhanced detection of quantum dots labeled protein by simultaneous bismuth electrodeposition into microfluidic channel. Electrophoresis.

[76] Freitas M, Nouws HP, Delerue-Matos C (2021). Voltammetric immunosensor to track a major peanut allergen (Ara h 1) in food products employing quantum dot labels. Biosensors.

[77] Álvarez J (2014). Real time optical immunosensing with flow-through porous alumina membranes. Sens. Actuators B Chem..

[78] Märki I, Rebeaud F (2017). Nanotechnologies for in vitro IgE testing. Curr. Allergy Asthma Rep..

[79] Mousavi SM (2021). Recent biotechnological approaches for treatment of novel COVID-19: from bench to clinical trial. Drug Metab. Rev..

[80] Omidifar, N. et al. Different laboratory diagnosis methods of covid-19: a systematic review. *Arch. Clin. Infect. Dis.* **16**, 1–12 (2021).

[81] Shi G-F (2015). An electrochemiluminescence aptasensor based on flowerlike CdS–MoS_2_ composites and DNAzyme for detection of immunoglobulin E. Sens. Actuators B Chem..

[82] Liu Y-M (2016). An electrochemiluminescence aptasensor based on CdSe/ZnS functionalized MoS2 and enzymatic biocatalytic precipitation for sensitive detection of immunoglobulin E. Sens. Actuators B Chem..

[83] Su S (2019). Two-dimensional nanomaterials for biosensing applications. Trends Anal. Chem..

[84] Pfender, N. et al. Evaluation of a Novel Rapid Test System for the Detection of Specific IgE to Hymenoptera Venoms. *J. Allergy (Cairo)*. **2012**, 862023 (2012). 10.1155/2012/862023 PMC3303699 22500188

[85] Oleinikov V (2011). Fluorescent semiconductor nanocrystals (quantum dots) in protein biochips. Rus. J. Bioorgan. Chem..

[86] Wang Y, Bai Y, Wei X (2011). Conjugation behaviours of CdTe quantum dots and antibody by a novel immunochromatographic method. IET Nanobiotechnol..

[87] Cronin L (2014). An in vitro study of the photodynamic effect of rose bengal on *Trichophyton rubrum*. J. Biophoton..

[88] Sadraeian M (2021). Photoinduced photosensitizer–antibody conjugates kill HIV env-expressing cells, also inactivating HIV. ACS Omega.

[89] Sadraeian M, Zhang L, Aavani F, Biazar E, Jin D (2022). Photodynamic viral inactivation assisted by photosensitizers. Mater. Today Phys..

[90] Zhao Y (2017). Quantum dots-based lateral flow immunoassay combined with image analysis for semiquantitative detection of IgE antibody to mite. Int. J. Nanomed..

[91] Koren A (2020). Fluorescent labeling of major honeybee allergens Api m 1 and Api m 2 with quantum dots and the development of a multiplex basophil activation test. Allergy.

[92] Aquino A, Conte-Junior CA (2020). A systematic review of food allergy: nanobiosensor and food allergen detection. Biosensors.

[93] Sadraeian M (2022). Study of viral photoinactivation by UV-C light and photosensitizer using a pseudotyped model. Pharmaceutics.

[94] He S, Li X, Gao J, Tong P, Chen H (2018). Development of a H_2_O_2_‐sensitive quantum dots‐based fluorescent sandwich ELISA for sensitive detection of bovine β‐lactoglobulin by monoclonal antibody. J. Sci. Food Agr..

[95] Song Q (2022). Quantum dot nanobead-based fluorescence-linked immunosorbent assay for detection of glycinin in soybeans and soy products. Molecules.

[96] Wang Y (2021). A sensitive immunosensor based on FRET between gold nanoparticles and InP/ZnS quantum dots for arginine kinase detection. Food Chem..

[97] Nurmatov U (2017). Allergen immunotherapy for IgE‐mediated food allergy: a systematic review and meta‐analysis. Allergy.

[98] Pfaar O (2019). Perspectives in allergen immunotherapy: 2019 and beyond. Allergy.

[99] Pajno GB (2018). EAACI Guidelines on allergen immunotherapy: IgE‐mediated food allergy. Allergy.

[100] Pfaar O, Lou H, Zhang Y, Klimek L, Zhang L (2018). Recent developments and highlights in allergen immunotherapy. Allergy.

[101] Han C (2021). Duplex metal co-doped carbon quantum dots-based drug delivery system with intelligent adjustable size as adjuvant for synergistic cancer therapy. Carbon.

[102] Cheng J (2019). Novel carbon quantum dots can serve as an excellent adjuvant for the gp85 protein vaccine against avian leukosis virus subgroup J in chickens. Poult. Sci..

[103] Jensen-Jarolim E, Roth-Walter F, Jordakieva G, Pali-Schöll I (2021). Allergens and adjuvants in allergen immunotherapy for immune activation, tolerance, and resilience. J. Allergy Clin. Immunol. Pract..

[104] Zubeldia J, Ferrer M, Dávila I, Justicia J (2019). Adjuvants in allergen-specific immunotherapy: modulating and enhancing the immune response. J. Investig. Allergol. Clin. Immunol..

[105] Alsaleh NB, Brown JM (2020). Engineered nanomaterials and type I allergic hypersensitivity reactions. Front. Immunol..

[106] Jatana S, Palmer BC, Phelan SJ, DeLouise LA (2017). Immunomodulatory effects of nanoparticles on skin allergy. Sci. Rep..

[107] Verlaet AA (2019). Oxidative stress and immune aberrancies in attention-deficit/hyperactivity disorder (ADHD): a case–control comparison. Eur. Child. Adolesc. Psychiatry.

[108] Gao N (2021). Defective Ag–In–S/ZnS quantum dots: an oxygen-derived free radical scavenger for mitigating macrophage inflammation. J. Mater. Chem. B.

[109] Kong H (2021). The bioactivity of scutellariae radix carbonisata-derived carbon dots: antiallergic effect. J. Biomed. Nanotechnol..

[110] Gidwani B (2021). Quantum dots: prospectives, toxicity, advances and applications. J. Drug Deliv. Sci. Technol..

[111] Yong K-T (2013). Nanotoxicity assessment of quantum dots: from cellular to primate studies. Chem. Soc. Rev..

[112] Das, K. et al. Bioaccumulation of CdSe quantum dots show biochemical and oxidative damage in Wistar rats. *Oxid. Med. Cell. Longev.* **2023**, 1–13 (2023). 10.1155/2023/7707452 PMC10101743 37064800

[113] Wu T, Tang M (2014). Toxicity of quantum dots on respiratory system. Inhal. Toxicol..

[114] Lv W (2019). Improving the stability of metal halide perovskite quantum dots by encapsulation. Adv. Mater..

[115] Alibolandi M (2016). Folate receptor-targeted multimodal polymersomes for delivery of quantum dots and doxorubicin to breast adenocarcinoma: in vitro and in vivo evaluation. Int. J. Pharmaceut..

[116] Javanbakht S, Shaabani A (2019). Encapsulation of graphene quantum dot-crosslinked chitosan by carboxymethylcellulose hydrogel beads as a pH-responsive bio-nanocomposite for the oral delivery agent. Int. J. Biol. Macromol..

[117] Son YR, Kwak M, Lee S, Kim HS (2020). Strategy for encapsulation of CdS quantum dots into zeolitic imidazole frameworks for photocatalytic activity. Nanomaterials.

[118] Wang Q, Chao Y (2018). Multifunctional quantum dots and liposome complexes in drug delivery. J. Biomed. Res..

[119] Probst CE, Zrazhevskiy P, Bagalkot V, Gao X (2013). Quantum dots as a platform for nanoparticle drug delivery vehicle design. Adv. drug Deliv. Rev..

[120] Pham X-H (2021). Synthesis and application of silica-coated quantum dots in biomedicine. Int. J. Mol. Sci..

[121] Liang Z, Khawar MB, Liang J, Sun H (2021). Bio-conjugated quantum dots for cancer research: detection and imaging. Front. Oncol..

[122] Fatima I (2021). Quantum dots: synthesis, antibody conjugation, and HER2-receptor targeting for breast cancer therapy. J. Funct. Biomater..

[123] Souza SO (2021). Methods for intracellular delivery of quantum dots. Top. Curr. Chem..

[124] Zhou M, Ghosh I (2007). Quantum dots and peptides: a bright future together. Biopolymers.

[125] Davodabadi, F. et al. Aptamer‐functionalized quantum dots as theranostic nanotools against cancer and bacterial infections: a comprehensive overview of recent trends. *Biotechnol. Prog.* **39**, e3366 (2023). 10.1002/btpr.3366 37222166

[126] Kim D, Yoo S (2021). Aptamer-conjugated quantum dot optical biosensors: strategies and applications. Chemosensors.

[127] Sharma A, Das J (2019). Small molecules derived carbon dots: synthesis and applications in sensing, catalysis, imaging, and biomedicine. J. Nanobiotechnol..

[128] Zia-ur-Rehman, M. et al. Recent Progress of Nanotoxicology in Plants. *Algae, and Microorganisms*. **1**, 143–174 (2018).

[129] Handlogten MW, Kiziltepe T, Serezani AP, Kaplan MH, Bilgicer B (2013). Inhibition of weak-affinity epitope-IgE interactions prevents mast cell degranulation. Nat. Chem. Biol..

[130] Wang Y, Tang M (2018). Review of in vitro toxicological research of quantum dot and potentially involved mechanisms. Sci. Total Environ..

[131] Hu L, Zhong H, He Z (2019). The cytotoxicities in prokaryote and eukaryote varied for CdSe and CdSe/ZnS quantum dots and differed from cadmium ions. Ecotox. Environ. Safe.

[132] Liang Y, Zhang T, Tang M (2022). Toxicity of quantum dots on target organs and immune system. J. Appl. Toxicol..

[133] Hu L, Zhong H, He Z (2021). Toxicity evaluation of cadmium-containing quantum dots: a review of optimizing physicochemical properties to diminish toxicity. Colloid. Surf. B.

[134] Liu N, Tang M (2020). Toxicity of different types of quantum dots to mammalian cells in vitro: an update review. J. Hazard Mater..

[135] Stan MS (2014). Si/SiO2 quantum dots cause cytotoxicity in lung cells through redox homeostasis imbalance. Chem. Biol. Interact..

[136] Tuncer Degim I, Kadioglu D (2013). Cheap, suitable, predictable and manageable nanoparticles for drug delivery: quantum dots. Curr. Drug Deliv..

[137] Zhao M-X, Zhu B-J (2016). The research and applications of quantum dots as nano-carriers for targeted drug delivery and cancer therapy. Nanoscale Res. Lett..

[138] Ye, L. et al. in *Nano-Enabled Medical Applications.* 431–455 (Jenny Stanford Publishing, 2020).

[139] Bachtold A (2000). Scanned probe microscopy of electronic transport in carbon nanotubes. Phys. Rev. Lett..

[140] Huang X, Tang M (2021). Review of gut nanotoxicology in mammals: Exposure, transformation, distribution and toxicity. Sci. Total Environ..

[141] Wang Z, Tang M (2021). Research progress on toxicity, function, and mechanism of metal oxide nanoparticles on vascular endothelial cells. J. Appl. Toxicol..

[142] Zhang LW, Bäumer W, Monteiro-Riviere NA (2011). Cellular uptake mechanisms and toxicity of quantum dots in dendritic cells. Nanomedicine.

[143] Belinova T (2018). Silicon quantum dots and their impact on different human cells. Phys. Status Solidi B.

[144] Mousavi, S. M. et al. Bioinorganic synthesis of polyrhodanine stabilized Fe_3_O_4_/Graphene oxide in microbial supernatant media for anticancer and antibacterial applications. *Bioinorg. Chem. Appl.* **2021**, 1–12 (2021). 10.1155/2021/9972664 PMC8257353 34257633

[145] Fucikova J, Palova-Jelinkova L, Bartunkova J, Spisek R (2019). Induction of tolerance and immunity by dendritic cells: mechanisms and clinical applications. Front. Immunol..

[146] Raker VK, Domogalla MP, Steinbrink K (2015). Tolerogenic dendritic cells for regulatory T cell induction in man. Front. Immunol..

[147] Hubo M (2013). Costimulatory molecules on immunogenic versus tolerogenic human dendritic cells. Front. Immunol..

[148] Wang X (2016). Immunotoxicity assessment of CdSe/ZnS quantum dots in macrophages, lymphocytes and BALB/c mice. J. Nanobiotechnol.

[149] Wu T (2019). The role of NLRP3 inflammasome activation in the neuroinflammatory responses to Ag 2 Se quantum dots in microglia. Nanoscale.

[150] Bhattacharya A (2015). Autophagy is required for neutrophil-mediated inflammation. Cell Rep..

[151] Zhang Y (2013). Functionalized quantum dots induce proinflammatory responses in vitro: the role of terminal functional group-associated endocytic pathways. Nanoscale.

[152] Qin Y (2015). Graphene quantum dots induce apoptosis, autophagy, and inflammatory response via p38 mitogen-activated protein kinase and nuclear factor-κB mediated signaling pathways in activated THP-1 macrophages. Toxicology.

[153] Lee B-C (2020). Graphene quantum dots as anti-inflammatory therapy for colitis. Sci. Adv..

[154] Tomić S (2017). Graphene quantum dots suppress proinflammatory T cell responses via autophagy-dependent induction of tolerogenic dendritic cells. Biomaterials.

[155] Klapper Y (2015). Low affinity binding of plasma proteins to lipid-coated quantum dots as observed by in situ fluorescence correlation spectroscopy. Nanoscale.

[156] Li S, Guo Z, Zhang Y, Xue W, Liu Z (2015). Blood compatibility evaluations of fluorescent carbon dots. ACS Appl. Mater. Interfaces.

[157] Deng S (2018). Transcriptomic response and perturbation of toxicity pathways in zebrafish larvae after exposure to graphene quantum dots (GQDs). J. Hazard Mater..

[158] Luo Y-H (2013). Cadmium-based quantum dot induced autophagy formation for cell survival via oxidative stress. Chem. Res. Toxicol..

[159] Seleverstov O (2006). Quantum dots for human mesenchymal stem cells labeling. a size-dependent autophagy activation. Nano Lett..

[160] Volarevic V (2014). Large graphene quantum dots alleviate immune-mediated liver damage. ACS Nano.

[161] Fan J (2016). Inhibition of autophagy overcomes the nanotoxicity elicited by cadmium-based quantum dots. Biomaterials.

[162] Cao X (2016). Self-regulation and cross-regulation of pattern-recognition receptor signalling in health and disease. Nat. Rev. Immunol..

[163] Nguyen KC (2019). Biodistribution and systemic effects in mice following intravenous administration of cadmium telluride quantum dot nanoparticles. Chem. Res. Toxicol..

[164] Zhu C (2019). Recent advances in non-toxic quantum dots and their biomedical applications. Prog. Nat. Sci.: Mater. Int..

[165] Chen N (2012). The cytotoxicity of cadmium-based quantum dots. Biomaterials.

[166] Dubertret B (2002). In vivo imaging of quantum dots encapsulated in phospholipid micelles. Science.

[167] Gao H (2017). Microwave assisted synthesis of luminescent carbonaceous nanoparticles from silk fibroin for bioimaging. Mater. Sci. Eng.: C..

[168] Qi K, Wang Y, Wang R, Wu D, Li G-D (2016). Facile synthesis of homogeneous CuInS 2 quantum dots with tunable near-infrared emission. J. Mater. Chem. C..

[169] Oda M, Miyaoka T, Yamada S, Tani T (2012). Synthesis, characterization and its photoluminescence properties of group I-III-VI2 CuInS2 nanocrystals. Phys. Procedia.

[170] Abbas A, Mariana LT, Phan AN (2018). Biomass-waste derived graphene quantum dots and their applications. Carbon.

[171] Kalashgrani MY, Nejad FF, Rahmanian V (2022). Carbon quantum dots platforms: as nano therapeutic for biomedical applications. Adv. Appl. NanoBio-Technol..

[172] Khan, A., Ezati, P., Kim, J.-T. & Rhim, J.-W. Biocompatible carbon quantum dots for intelligent sensing in food safety applications: opportunities and sustainability. *Mater. Today Sustain.* **21**, 100306 (2022).

[173] Umar, E. et al. A state-of-the-art review on carbon quantum dots: prospective, advances, zebrafish biocompatibility and bioimaging in vivo and bibliometric analysis. *Sustain. Mater. Technol.* **35**, e00529 (2022).

[174] Bhandari S, Mondal D, Nataraj S, Balakrishna RG (2019). Biomolecule-derived quantum dots for sustainable optoelectronics. Nanoscale Adv..

[175] Zheng XT, Lai YC, Tan YN (2019). Nucleotide-derived theranostic nanodots with intrinsic fluorescence and singlet oxygen generation for bioimaging and photodynamic therapy. Nanoscale Adv..

[176] Torres-Huerta AL, Antonio-Pérez A, García-Huante Y, Alcázar-Ramírez NJ, Rueda-Silva JC (2022). Biomolecule-based optical metamaterials: design and applications. Biosensors.

[177] Ananthanarayanan A (2015). Nitrogen and phosphorus co-doped graphene quantum dots: synthesis from adenosine triphosphate, optical properties, and cellular imaging. Nanoscale.

[178] Jha A (2020). DNA biodots based targeted theranostic nanomedicine for the imaging and treatment of non-small cell lung cancer. Int. J. Biol. Macromolecules.

[179] Tavakkoli Yaraki M, Liu B, Tan YN (2022). Emerging strategies in enhancing singlet oxygen generation of nano-photosensitizers toward advanced phototherapy. Nano-Micro Lett..

